# EASY‐APP: An artificial intelligence model and application for early and easy prediction of severity in acute pancreatitis

**DOI:** 10.1002/ctm2.842

**Published:** 2022-06-02

**Authors:** Balázs Kui, József Pintér, Roland Molontay, Marcell Nagy, Nelli Farkas, Noémi Gede, Áron Vincze, Judit Bajor, Szilárd Gódi, József Czimmer, Imre Szabó, Anita Illés, Patrícia Sarlós, Roland Hágendorn, Gabriella Pár, Mária Papp, Zsuzsanna Vitális, György Kovács, Eszter Fehér, Ildikó Földi, Ferenc Izbéki, László Gajdán, Roland Fejes, Balázs Csaba Németh, Imola Török, Hunor Farkas, Artautas Mickevicius, Ville Sallinen, Shamil Galeev, Elena Ramírez‐Maldonado, Andrea Párniczky, Bálint Erőss, Péter Jenő Hegyi, Katalin Márta, Szilárd Váncsa, Robert Sutton, Peter Szatmary, Diane Latawiec, Chris Halloran, Enrique de‐Madaria, Elizabeth Pando, Piero Alberti, Maria José Gómez‐Jurado, Alina Tantau, Andrea Szentesi, Péter Hegyi, the Hungarian Pancreatic Study Group

**Affiliations:** ^1^ Department of Medicine University of Szeged Szeged Hungary; ^2^ Centre for Translational Medicine, Department of Medicine University of Szeged Szeged Hungary; ^3^ Department of Stochastics, Institute of Mathematics Budapest University of Technology and Economics Budapest Hungary; ^4^ MTA‐BME Stochastics Research Group Budapest Hungary; ^5^ Institute for Translational Medicine, Szentágothai Research Centre, Medical School University of Pécs Pécs Hungary; ^6^ Institute of Bioanalysis, Medical School University of Pécs Pécs Hungary; ^7^ Division of Gastroenterology, First Department of Medicine University of Pécs, Medical School Pécs Hungary; ^8^ Department of Gastroenterology, Institute of Internal Medicine, Faculty of Medicine University of Debrecen Debrecen Hungary; ^9^ Szent György Teaching Hospital of County Fejér Székesfehérvár Hungary; ^10^ County Emergency Clinical Hospital of Târgu Mures—Gastroenterology Clinic and University of Medicine, Pharmacy, Sciences and Technology ‘George Emil Palade’ Targu Mures Romania; ^11^ Vilnius University Hospital Santaros Clinics Vilnius Lithuania; ^12^ Department of Transplantation and Liver Surgery Helsinki University Hospital and University of Helsinki Helsinki Finland; ^13^ Saint Luke Clinical Hospital St. Petersburg Russia; ^14^ Department of General Surgery Consorci Sanitari del Garraf Sant Pere de Ribes Spain; ^15^ Heim Pál National Pediatric Institute Budapest Hungary; ^16^ Division of Pancreatic Diseases, Heart and Vascular Centre Semmelweis University Budapest Hungary; ^17^ Centre for Translational Medicine Semmelweis University Budapest Hungary; ^18^ Institute of Systems, Molecular and Integrative Biology University of Liverpool and Liverpool University Hospitals NHS Foundation Trust, Liverpool, England UK; ^19^ Gastroenterology Department Alicante University General Hospital ISABIAL Alicante Spain; ^20^ Department of Hepato‐Pancreato‐Biliary and Transplant Surgery Hospital Universitari Vall d'Hebron, Universitat Autònoma de Barcelona Barcelona Spain; ^21^ The 4th Medical Clinic Iuliu Hatieganu’ University of Medicine and Pharmacy Cluj‐Napoca Romania; ^22^ Gastroenterology and Hepatology Medical Center Cluj‐Napoca Romania

**Keywords:** acute pancreatitis, artificial intelligence, severity prediction

## Abstract

**Background:**

Acute pancreatitis (AP) is a potentially severe or even fatal inflammation of the pancreas. Early identification of patients at high risk for developing a severe course of the disease is crucial for preventing organ failure and death. Most of the former predictive scores require many parameters or at least 24 h to predict the severity; therefore, the early therapeutic window is often missed.

**Methods:**

The early achievable severity index (EASY) is a multicentre, multinational, prospective and observational study (ISRCTN10525246). The predictions were made using machine learning models. We used the scikit‐learn, xgboost and catboost Python packages for modelling. We evaluated our models using fourfold cross‐validation, and the receiver operating characteristic (ROC) curve, the area under the ROC curve (AUC), and accuracy metrics were calculated on the union of the test sets of the cross‐validation. The most critical factors and their contribution to the prediction were identified using a modern tool of explainable artificial intelligence called SHapley Additive exPlanations (SHAP).

**Results:**

The prediction model was based on an international cohort of 1184 patients and a validation cohort of 3543 patients. The best performing model was an XGBoost classifier with an average AUC score of 0.81 ± 0.033 and an accuracy of 89.1%, and the model improved with experience. The six most influential features were the respiratory rate, body temperature, abdominal muscular reflex, gender, age and glucose level. Using the XGBoost machine learning algorithm for prediction, the SHAP values for the explanation and the bootstrapping method to estimate confidence, we developed a free and easy‐to‐use web application in the Streamlit Python‐based framework (http://easy‐app.org/).

**Conclusions:**

The EASY prediction score is a practical tool for identifying patients at high risk for severe AP within hours of hospital admission. The web application is available for clinicians and contributes to the improvement of the model.

## Graphical abstract

- The EASY prediction score is a practical tool for identifying patients at a greater risk for severe acute pancreatitis shortly after hospital admission.
- The explanation of the impact of features on the prediction helps physicians understand the decision of the machine learning model.
- The easy‐to‐use web application is available for clinicians and contributes to the improvement of the model.

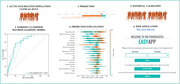

## 1 INTRODUCTION

Acute pancreatitis (AP) is one of the most challenging and common gastroenterological diseases that requires hospitalisation. The importance of the investigation of AP is uncontroversial: more than 2.6 billion dollars is the annual cost of the treatment of AP in the USA, where it causes approximately 300 000 emergency department visits every year.^1^, ^2^

According to the revised Atlanta classification, the severity of AP can be determined as mild, moderately severe and severe disease course.^3^ In general, 70%–75% of patients have mild disease with a very low mortality rate; however, the remaining 20%–25% of patients have moderately severe disease, and 5%–10% have severe disease with high mortality.^4^, ^5^ Moderately severe AP is associated with transient organ failure (less than 48 h) and/or local complications. In the case of severe AP, organ failure is persistent (more than 48 h), with a mortality rate up to 50%.^3^, ^6^ Mortality in AP is spread over two periods: during the early phase (first 2 weeks), indicative of rampant disease, or during the late phase (third week and later) following progressive deterioration.^7^, ^8^, ^9^

Early identification of those patients who are at a greater risk for developing complications is necessary to reduce the risk of adverse disease outcomes and death. Several prediction scores and biochemical markers have been evaluated and compared in the past.^10^, ^11^, ^12^, ^13^, ^14^, ^15^, ^16^ No laboratory test is consistently accurate for the prediction of AP severity. For example, C‐reactive protein (CRP) levels at 48 h are significantly higher in the severe pancreatitis group than in the others but cannot be used on admission because of the low accuracy.^17^, ^18^ Concerning multifactorial scoring systems, all have limitations: typically, these require many and/or not easily accessible variables or 48–72 h for evaluation. As a result, none has been adopted for widespread use in daily clinical practice. The Acute Physiology and Chronic Health Examination (APACHE) II score was developed for the assessment of critically ill patients, not specific to AP. The calculation of APACHE II is complicated and requires invasive measurements, including blood gases.^19^ Ranson and Glasgow‐Imrie scores contain parameters that are not routinely measured, and completion of these scores requires 48 h from hospital admission, losing critical time for more intensive resuscitation, analgesia and nutritional support.^20^, ^21^ More recently, developed scores for assessing the severity of AP are the Bedside Index of Severity in Acute Pancreatitis (BISAP) and the Harmless Acute Pancreatitis Score (HAPS). While the BISAP score was developed to predict severe AP and mortality, HAPS can predict non‐severe AP with high (96%–97%) sensitivity and positive predictive value (98%).^22^, ^23^ The Balthazar score and the newer computer tomography (CT) scores (mCTSI, SMART‐CT index) are useful for the characterisation of local injury but are largely useful only several days after admission and ignore clinical symptoms and signs.^24^, ^25^, ^26^

We can conclude that most earlier prognostic scores need at least 24 h to predict severity or that several parameters are not easily available on admission; therefore, early prediction of AP severity is still awaiting a solution.^18^, ^27^

Many attempts have been made to use artificial intelligence in healthcare, among others in radiology,^28^, ^29^ in pathology^30^ or for prediction, as it can detect complex nonlinear relationships between various biochemical parameters and disease outcomes.^11^, ^31^ As a type of artificial intelligence, a machine learning algorithm builds a model based on a training dataset and can improve its performance with experience. Several AP severity prediction models used artificial intelligence and machine learning, but they were based on datasets with low patient numbers and used only internal validation methods.^32^, ^33^, ^34^, ^35^

Our principal aim was to develop and validate a clinical prediction model of severity in AP that requires parameters easily accessible on admission. Our further aim was to design and develop a practical application for clinicians for the easy prediction of severe AP.

## 2 METHODS

### 2.1 Preliminary settings

The study protocol was discussed at the third meeting of the Hungarian Pancreatic Study Group (HPSG) in 2014, and the pre‐study protocol was published in 2015.^36^ Ethical permission was given by the Scientific and Research Ethics Committee of the Hungarian Medical Research Council (30595/2014/EKU), and the trial was registered in the international ISRCTN registry (ISRCTN10525246). The electronic clinical data registration (eCRF) system for data collection and management was developed and run by the HPSG.

### 2.2 Study design

The early achievable severity index (EASY) is a multinational, multicentre, prospective and observational study. In the first phase of the study, simple attainable parameters (medical history, anamnestic data, physical examination, laboratory parameters and imaging details) were recorded on hospital admission from AP patients from 15 countries and 28 medical centres. In the second phase of the study, validation data of AP patients were collected and analysed from four international pancreatic patient care and research centres. The centre distribution and case numbers are shown in Tables S1 and S2.

### 2.3 Population

AP patients 18 years of age or older assessed within 12 h of admission were enrolled after giving their informed consent. Both the definition of AP and severity were based on the revised Atlanta classification,^3^ and the ‘IAP/APA evidence‐based guidelines for the management of acute pancreatitis^37^’ should have been followed in the diagnosis and treatment of AP patients.

### 2.4 Data collection and management

According to the literature data of predictive scores (APACHE II, Glasgow‐Imrie, HAPS, BISAP), data of potential prognostic parameters (e.g., medical history, laboratory tests, physical examination and diagnostic imaging details) were collected. Abdominal ultrasound or contrast‐enhanced CT scan was used to determine pancreatic alterations (necrosis, fluid collections, pseudocyst formation, etc.) or chest X‐ray was performed to determine the pleural effusion of lung infiltration. These imaging diagnostic tools had standard protocols based on the regulation of radiological organisations. The evaluation of pancreas‐related local complications was based on the revised Atlanta classification.^3^, ^38^

We applied a four‐step data quality control system: after local administrative validation and local professional approval, a central administrative and professional check was undertaken by the study coordination team. Cases of insufficient data quality were excluded from the analysis. The flowchart of patient inclusion can be found in Figure S1.

### 2.5 Outcome

After classifying the population into severity groups according to the revised Atlanta classification, a composite binary label was constructed based on the dataset to define the severity of pancreatitis used in the model. The new label was 1 if the outcome of the disease was fatal or the AP was classified as severe (severe AP); otherwise, the label was 0 (non‐severe AP). This binary labelling methodology was used for both the original and validation cohorts.

### 2.6 Predictors and machine learning

Our goal was to predict whether a patient will develop severe or non‐severe AP (measured by the composite label introduced above) based on the data that are available at the time of hospital admission. In the language of data science, our problem is a binary classification problem, where the target variable (class label) is the binary degree of severity of AP. The explanatory variables are the parameters measured at the time of admission. We suppose that the reader is familiar with the basic concepts of machine learning and data science; otherwise, we refer to Rowe,^39^ where the author describes various concepts behind machine learning to make clinicians more familiar with these techniques.

There were two main challenges during data preparation: missing data and imbalanced class distribution. We used a *k*‐nearest‐neighbour‐based data imputer algorithm called KNNImputer^40^ to handle missing data. Since the dataset is highly imbalanced (only 6% of the patients were labelled severe), we applied the synthetic minority oversampling technique, the so‐called SMOTE algorithm,^41^ on the training set to oversample the severe cases. In the oversampled training dataset, the proportion of severe cases is 50%.

The predictions were made using several machine learning algorithms, including decision tree, random forest, logistic regression, support vector machine (SVM), CatBoost and XGBoost. The idea of using deep learning models was discarded due to the tabular nature of the data.^42^ For the modelling, we used the scikit‐learn,^43^ xgboost^44^ and catboost^45^ Python packages.

For the evaluation of the model, we used fourfold cross‐validation, which means that the data were divided into four equally sized subsets, and one of these subsets was selected as a test dataset, and the remaining data were used to train the machine learning model. The performance of the model is calculated on the selected test subset; then, this procedure is repeated for the other subsets as well, that is, in each round, one of the subsets is the test set, and the rest are the training data. Cross‐validation aggregates the performance metric, namely, it returns the average performance on the test sets.

We also evaluated our models using the receiver operating characteristic (ROC) curve, the corresponding area under the curve (AUC) score with its 95% confidence interval using bootstrapping,^46^ and accuracy metrics calculated from the union of the test sets of the cross‐validation.

**FIGURE 1:**
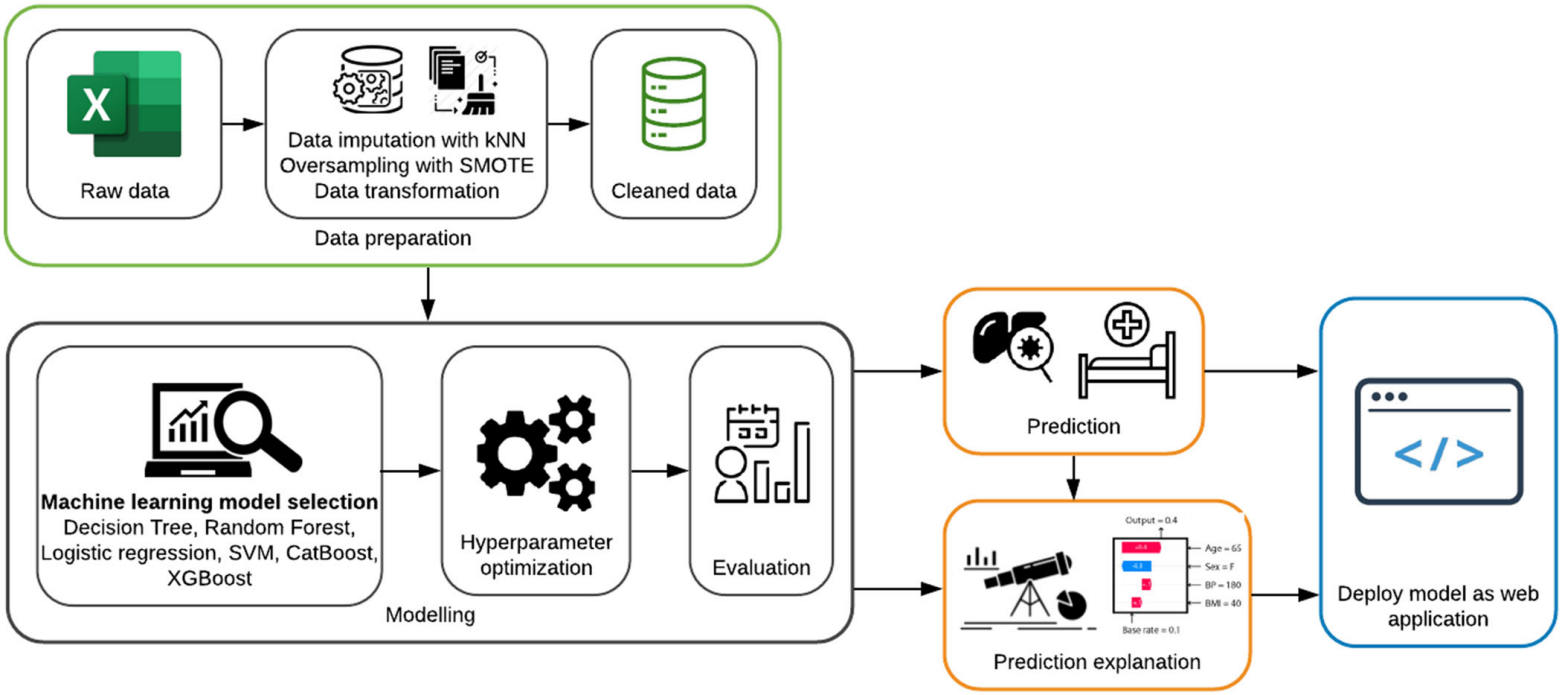
The workflow of the development of the prediction model

To measure the confidence of the model predictions, many copies of the machine learning model were trained using a bootstrapping method, that is, we resampled the training dataset 100 times and trained the copies of the model independently on these sets and calculated predicted scores. The 10th and 90th percentiles were used to construct a confidence interval over the score of the prediction. The workflow for developing the prediction model is shown in Figure 1.

### 2.7 Explaining the predictions

Besides predicting the severity of AP, another important goal is to identify the most important factors and their contribution to the prediction using a modern tool of explainable artificial intelligence called SHapley Additive exPlanations (SHAP).^47^ This so‐called SHAP value is able to explain the outcome of a machine learning model using a game‐theoretical concept: the Shapley value. The SHAP value quantifies the (marginal) contribution of each feature to the final prediction, which in our case is the severity score of AP. In other words, for a given feature *i*, the contribution of *i*, that is, its SHAP value, is the difference between the prediction using the value of *i* and the mean prediction.

Formally, let *f* denotes the machine learning that for given input parameters $x=(x_{1},\text{…},x_{d})$ of a patient returns the predicted severity score; moreover, let D={1,2,…,2} denotes the set of features. Then, let $f_{S}\left(x\right)$ be the conditional expectation of f(x) given the values of the features of the set *S*, that is, the values of $x_{i}$, where ∀i∈S. If *S* is an empty set, then $f_{S}\left(x\right)$ is the expectation of f(x); formally, $f_{\{\}Other}\;\left(x\right)=\;E\left(f(x)\right)$. Using these notations, let us define a function *v* that calculates the contribution of a feature set *S*: $v\left(S\right)=f_{S}\left(x\right)-f_{\left\{\right\}}\left(x\right)$, which is the difference between the prediction where we observe the values of the *S* subset of features and the mean prediction. The contribution $\varphi_{i}\left(x\right)$ of feature *i* for the prediction of *x* is defined using *v* as follows:

$$\varphi_{i}\left(x\right)=\sum_{S\subseteq D\setminus \{i\}}\frac{\left|S\right|!\left(d-\left|S\right|-1\right)!}{d!}\left(v(S\cup \{i\})-v(S)\right)$$

The SHAP feature importance $I_{j}$ of feature *j* is simply the mean absolute contribution of the feature where the average is taken on the whole dataset, that is:

$$\;I_{j}=\frac{1}{n}\sum_{i=1}^{n}\left|\varphi_{j}\left(x^{\left(i\right)}\right)\right|$$

Using the XGBoost machine learning algorithm for prediction, the SHAP values for the explanation, and the bootstrapping method for the estimation of confidence, we have developed a web application in the Streamlit Python‐based framework.

### 2.8 Statistical analyses

Case numbers and percentages were calculated for categorical variables, and means with standard deviations, minima and maxima were calculated for numerical variables in descriptive analyses of the original and validation cohorts. A two‐sided *p*‐value of < .05 was considered statistically significant.

## 3 RESULTS

### 3.1 Characteristics of the original cohort

A total of 1184 patients diagnosed with AP were included in the analysis. Eight hundred and seventy‐eight patients (74%) had mild, 243 (21%) moderately severe and 63 patients (5%) had a severe disease course according to the revised Atlanta classification. There were 26 deaths. With the constructed binary class label, 1114 patients (94%) were classified as non‐severe, while 70 patients (6%) were labelled as having severe disease. Hence, the data had a highly imbalanced class distribution. The general characteristics of the cohort are detailed in Table 1.

**TABLE 1:** Characteristics of the original cohort

			Data quality^a^
Demographic data			
Gender, male%	58.1%	Female/male	100%
Age, mean (SD); min, max	55.7 (16.6)	[17, 95]	100%
BMI, mean (SD); min, max	27.98 (5.86)	[14.8, 50.4]	99%
Anamnestic data			
Alcohol consumption, yes%	54.0%	Yes/no	100%
Smoking, yes%	34.4%	Yes/no	100%
Length of abdominal pain, mean (SD) in hours; min, max	36.8 (40.4)	[1, 168]	98%
Admission data			
Abdominal guarding, yes%	22.7%	Yes/no	99%
Abdominal tenderness, yes%	89.6%	Yes/no	99%
Body temperature (axillary),°C mean (SD); min, max	36.7 (0.46)	[34.8, 39.0]	98%
Systolic blood pressure (Hgmm), mean (SD); min, max	141.9 (22.5)	[75, 220]	100%
Diastolic blood pressure (Hgmm), mean (SD); min, max	85.2 (14.3)	[40, 191]	100%
Heart rate, mean (SD); min, max	83.9 (16.5)	[41, 153]	100%
Respiratory rate, mean (SD); min, max	17.7 (3.7)	[10, 45]	99%
Laboratory parameters			
Amylase (U/L), mean (SD); min, max	1077 (1117)	[16, 8544]	100%
Aspartate transaminase (U/L), mean (SD); min, max	147.9 (186)	[4, 1251]	99%
Serum ionized Calcium (mmol/L), mean (SD); min, max	2.31 (0.22)	[1.5, 4.5]	98%
C‐reactive protein (mg/L), mean (SD); min, max	49.76 (74.5)	[0.07, 515]	100%
Creatinine (μmol/L), mean (SD); min, max	85.8 (46.7)	[36, 706]	100%
Glucose (mmol/L), mean (SD); min, max	8.23 (3.48)	[2.53, 43.29]	100%
Potassium (mmol/L), mean (SD); min, max	4.12 (0.55)	[2.5, 7]	97%
Sodium (mmol/L), mean (SD); min, max	137.8 (4.1)	[116, 155]	97%
Urea (carbamide) (mmol/L), mean (SD); min, max	6.32 (3.85)	[0.98, 40.09]	100%
White blood cell count (G/L), mean (SD); min, max	12.78 (5.05)	[1.32, 52.70]	100%
Imaging examinations			
Pleural fluid	12.0%	Yes/no	88%
Acute peripancreatic fluid collection	22.2%	Yes/no	93%
Abdominal fluid	23.0%	Yes/no	96%
Outcome			
The severity of acute pancreatitis, severe%	5.9%	Non‐severe/severe	100%

Abbreviations: BMI, body mass index; SD, standard deviation.

^a^ Data not missing.

### 3.2 Machine learning models

**FIGURE 2:**
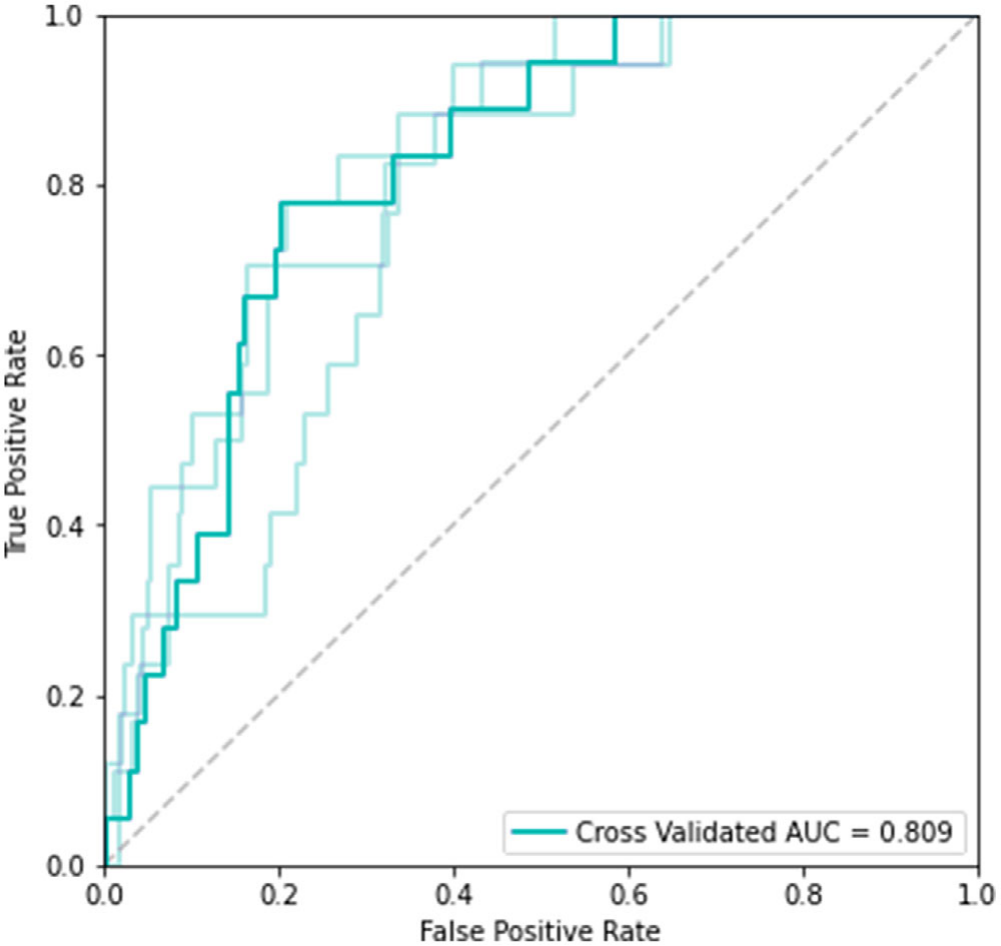
The cross‐validated (fold = 4) receiver operating characteristic (ROC) curve of the XGBoost model. The corresponding mean area under the curve (AUC) is 0.809. The 95% confidence interval is [0.776, 0.842]

We trained and evaluated the following binary classifiers: decision tree, random forest, logistic regression, SVM, CatBoost and XGBoost. The best performing model was an XGBoost classifier with an average AUC score of 0.81 ± 0.033 and an accuracy of 89.1%. The ROC curve and the corresponding AUC are depicted in Figure 2.

**FIGURE 3:**
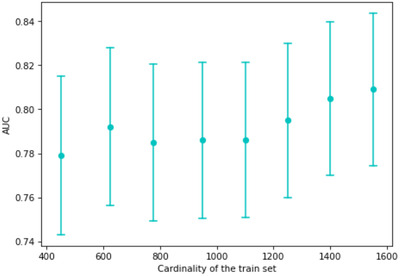
The performance of the XGBoost model trained on different sized sets. The points show the area under the curve (AUC) scores, and the bars are the corresponding confidence intervals

Although the size of our dataset is larger than that of previously published studies, we investigated how the performance of the model increases as we increase the size of the training set. We supposed that the model had not reached its maximal performance and could be further improved with more data (Figure 3).

**FIGURE 4:**
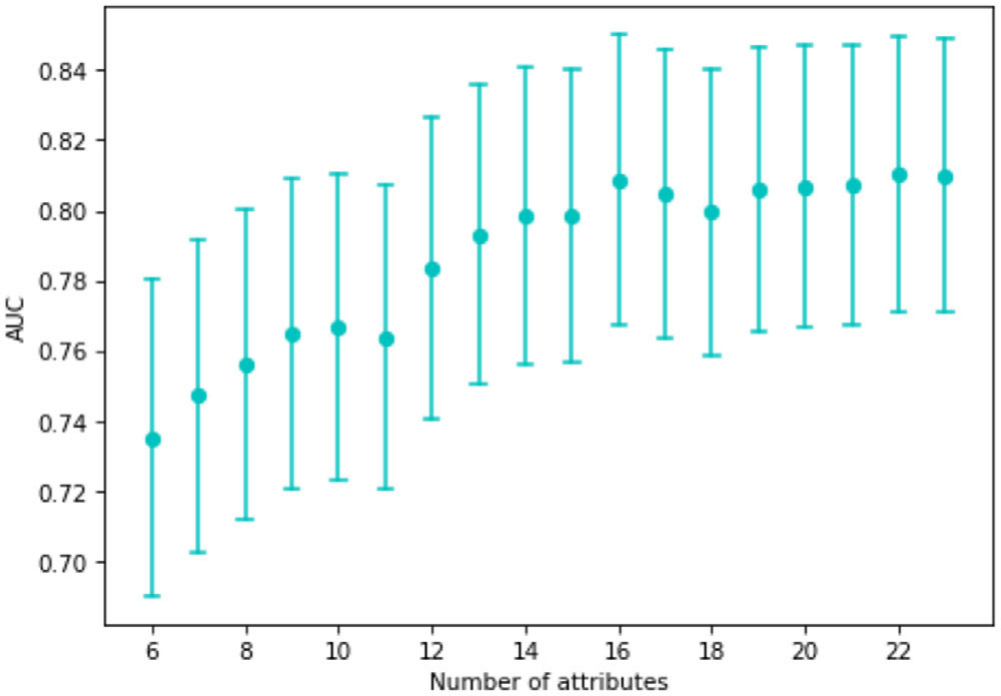
The performance of the model using varying numbers of attributes with the top *k* most important features. The importance is calculated using the SHapley Additive exPlanations (SHAP) importance. The points show the area under the curve (AUC) scores, and the bars are the corresponding confidence intervals

As not all parameters were measured or available on admission, we examined how the performance of the model decreases if it is built from fewer variables. The AUC values for the models built only on the most important *k* attributes (according to their SHAP values) are shown in Figure 4. It is clear that performance increased with the number of variables used for prediction, but reasonable performance was obtained with fewer parameters.

**FIGURE 5:**
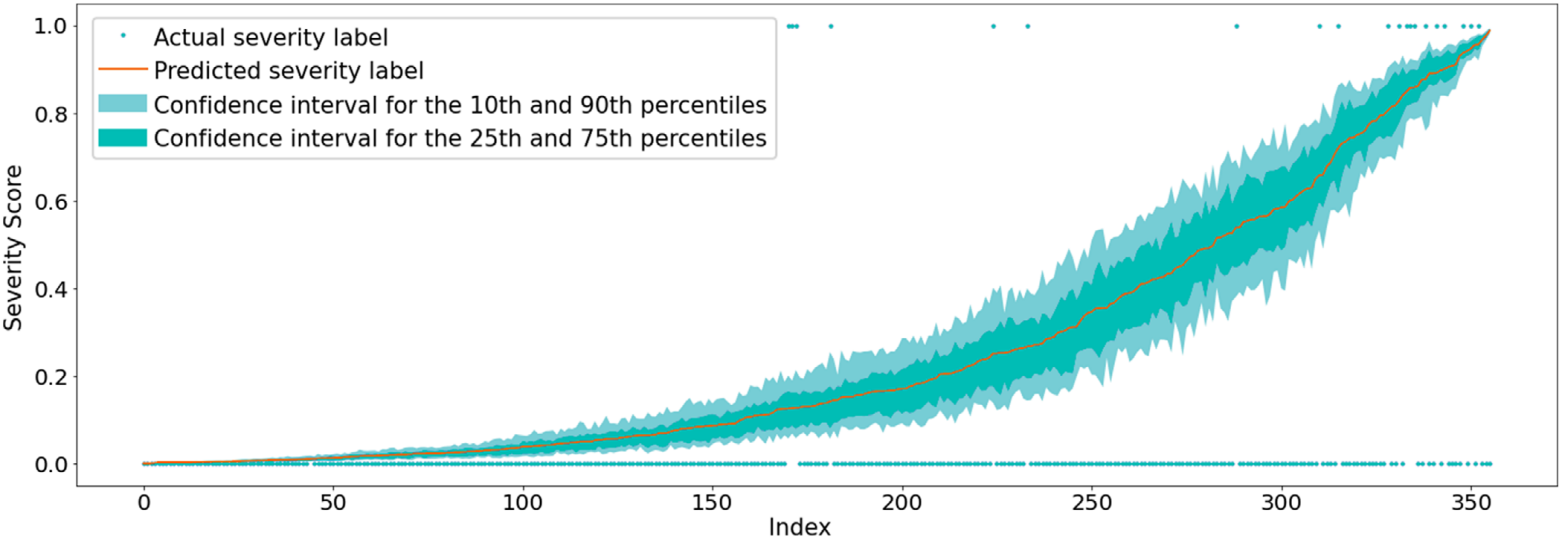
The predicted severity score on a selected subset of the dataset and the confidence intervals for the 10th and 90th percentiles and the 25th and 75th percentiles. The records are sorted by the severity score

For binary classification, machine learning models usually only predict a score that can be interpreted as the likelihood of the positive class, here the likelihood of severe AP. On the other hand, the confidence of the given prediction usually remains unclear. To assist the physicians in assessing to what extent they can rely on the model's output in decision‐making, we also estimated the confidence of the prediction using a bootstrapping method. The confidence intervals for a selected test dataset of 356 records (30% of the dataset) can be seen in Figure 5. The confidence of the model is greater near the endpoints of the spectrum, that is, when the degree of severity is clearly mild or severe. On the other hand, the width of the confidence intervals in the mid range is slightly larger.

### 3.3 Explaining the predictions

**FIGURE 6:**
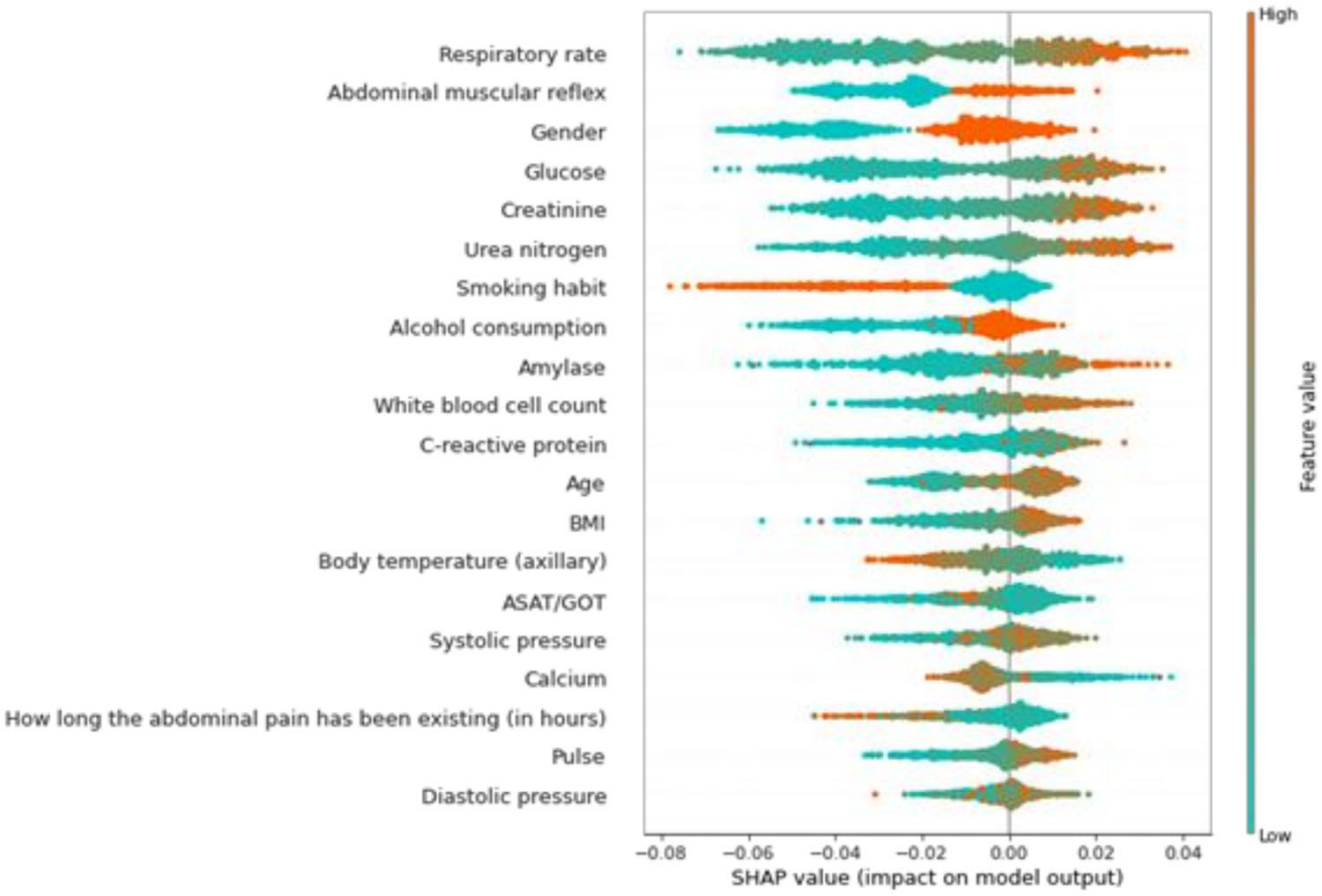
A summary plot of the impact of the features on the prediction (severity score) of the model. Each patient is represented by a point in each row. The colour of the points represents the relative value of the feature, and the *x*‐position of the points is the SHapley Additive exPlanations (SHAP) value, that is, the impact on the model's prediction

With the help of the SHAP values, the individual predictions of the machine learning model can be explained, and it is possible to measure the global importance of individual features. The effect of the individual features on the model output and their ranked importance are shown in Figure 6. The top six most influential features were respiratory rate, abdominal muscular reflex, gender, glucose, creatinine and urea nitrogen level. The most influential predictors slightly change if the model is trained on other validation cohorts. The six most influential features regarding all cohorts were creatinine, glucose, respiratory rate, urea nitrogen, white blood cell count and gender. More detail can be found in Table S9.

**FIGURE 7:**
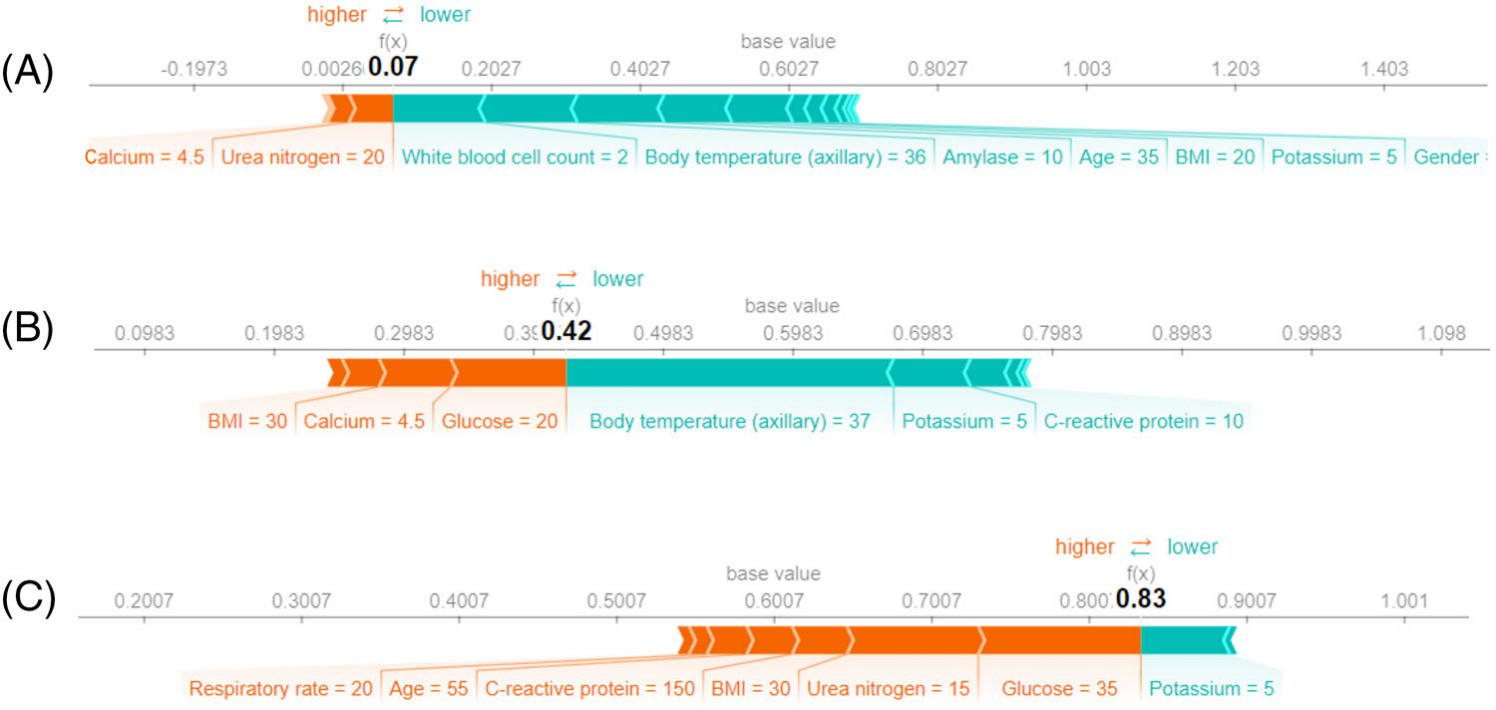
Three examples of the local explanation of the predictions using the SHapley Additive exPlanations (SHAP) values. (A) Predicted mild acute pancreatitis (AP). (B) Predicted AP with borderline severity. (C) Predicted severe AP. Factors that push the predicted score higher compared to the base value (mean prediction) are coloured orange, and those pushing lower the prediction are shown in green

Using the SHAP values, explanations of three different predictions are depicted in Figure 7. The features pushing higher the predicted probability of severe AP (compared to the mean prediction, called base value) are shown in orange, and those pushing the prediction lower are shown in green. Moreover, the length of the bars is proportional to the extent to which the corresponding factor contributes to the prediction. Note that due to oversampling, the average prediction on the training set does not reflect the prevalence of severe disease. Hence, it is not the exact SHAP value of a variable that is meaningful in a clinical setting but rather its sign and its relative value in comparison with the other variables’ SHAP value.

If most parameters of the patient are normal, the risk of developing severe AP is very low (Figure 7A). The fact that the body mass index (BMI) and glucose level are high, pushes the predicted severity score higher (Figure 7B). In the case of most parameters being outside the normal range (the patient was older and had a high glucose level, urea nitrogen, BMI, CRP and respiratory rate), the probability of severe disease increased (Figure 7C). More examples can be found in Figure S2.

### 3.4 Validation of the results

Our results were validated on external data from four international centres: Alicante, Barcelona, Cluj‐Napoca and Liverpool. Altogether, 3164 cases were included in the analysis. First, we validated the model's performance by training it on the EASY dataset, and then we measured its performance on the four international centres. The AUC scores of the model on the Alicante, Barcelona, Cluj‐Napoca and Liverpool data are 0.72 ± 0.036, 0.79 ± 0.039, 0.74 ± 0.041 and 0.77 ± 0.040, respectively. We found that the performance of the model improves significantly if we supplement the training data with the international datasets; in this case, the cross‐validated AUC score is 0.82 ± 0.011 on the EASY dataset, 0.79 ± 0.014 on the Alicante dataset, 0.82 ± 0.020 on the Barcelona dataset, 0.79 ± 0.023 on the Cluj‐Napoca dataset and 0.78 ± 0.026 on the Liverpool dataset. Finally, we measured the model's performance on the union of all the datasets; in this case, the cross‐validated AUC score was 0.803 ± 0.010. Further details of the validation cohort and the results of the analysis are available in Supporting Information.

### 3.5 Web application

Using the XGBoost machine learning algorithm for prediction, the SHAP values for the explanation, and the bootstrapping method for the estimation of confidence, we have developed a web application (http://easy‐app.org/) in the Streamlit Python‐based framework. The application is able to operate if not all the input variables are given; however, at least five input parameters have to be provided. Although XGBoost can handle missing data, to interpret the SHAP values, we solved this challenge by retraining the model using only the given parameters.

**FIGURE 8:**
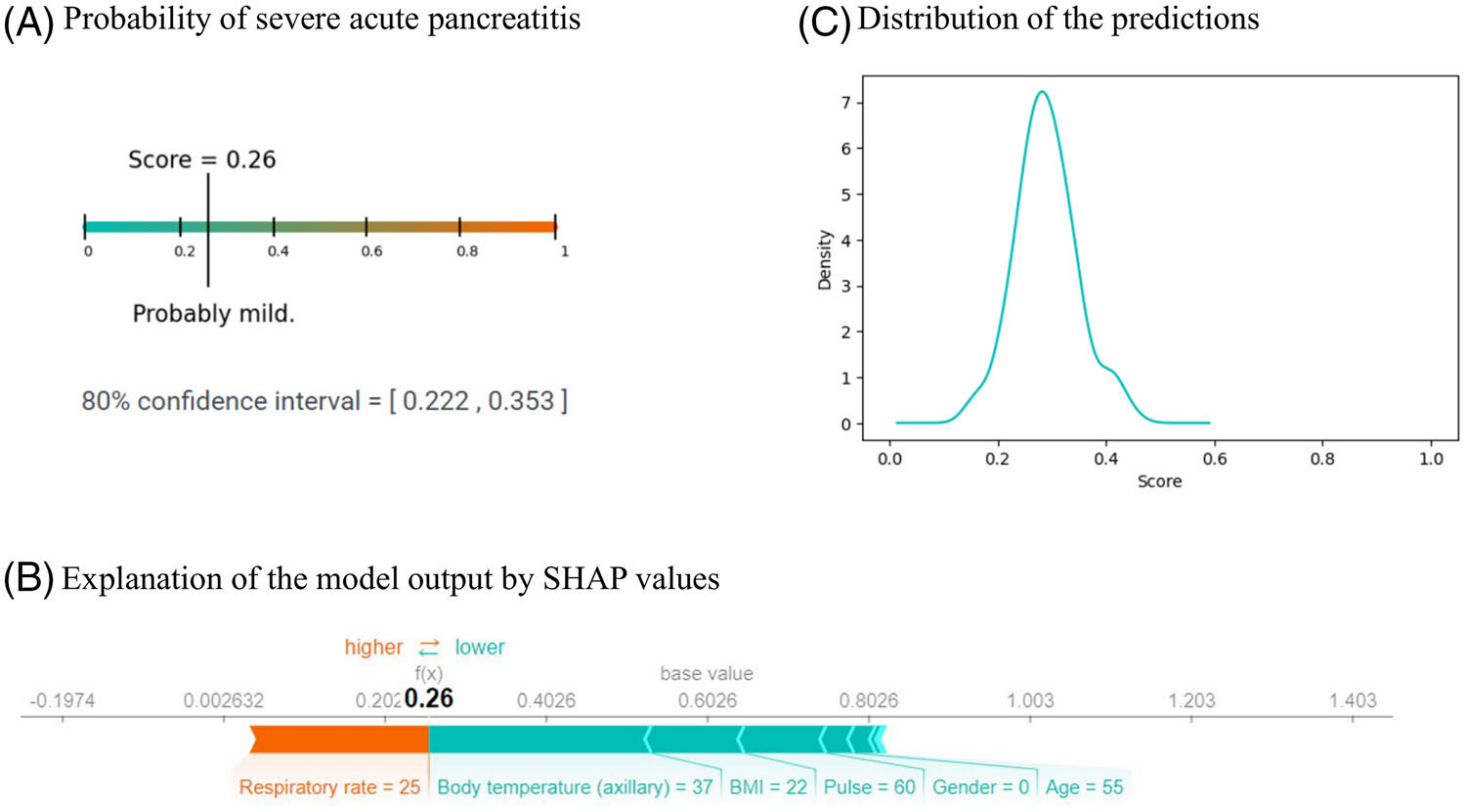
An example output of the web application for the following input parameters—age: 55 years, gender: 0 (woman), body mass index: 22, alcohol consumption: 1 (true), blood pressure/pulse: 140/75/60, body temperature: 37.0°C, respiratory rate: 25. (A) Predicted severity score. (B) Explanation of prediction. (C) The kernel density estimate plot of the distribution of the predictions

The application returns three different plots that show the probability of having severe pancreatitis according to the model (the predicted severity score), the confidence interval of the prediction severity score, the explanation of the decision of the model, and the distribution of the predicted scores made by the XGBoost models. A prediction in the application is shown in Figure 8.

## 4 DISCUSSION

We have applied machine learning to the development and testing of a simple risk score for severe AP between 0 and 1 that can be calculated on admission from simple bedside parameters. This score has been derived and validated by a study of almost 5000 patients from multiple countries, confirming its applicability at the bedside, which is now easily used in our web‐based application. Furthermore, it is expected that the score will improve with use as more data are entered. While machine learning models usually lack an explanation behind the output, operating as a ‘black box’,^11^ we solved the problem of machine learning model interpretation by applying a novel explainable artificial intelligence (XAI) tool, called SHAP value.^47^ This state‐of‐the‐art technique enabled us to identify the variables that affect the prediction, determining the most important factors and their contribution to the prediction. The power of SHAP values has also been illustrated by Lundberg et al.^48^ and Haimovich et al.^49^ who developed an early prognostic tool for the severity of COVID‐19 and used SHAP values to investigate the effects of the individual variables. To the best of our knowledge, this is the first work using SHAP values in the prediction of AP severity. In the EASY population, the most relevant factors causing more severe disease were respiratory rate, abdominal guarding, axillary body temperature, serum amylase, gender and serum glucose level, all routinely and easily obtained. From this, we have developed an easy‐to‐use web application that gives a prediction for the likelihood of severe AP using a given input of available parameters while explaining the prediction of the machine learning model, making it useful not only for prediction but also for education.

Handcrafted AP severity prediction scores (APACHE II, Ranson, Glasgow‐Imrie, CTSI, HAPS, BISAP and others) are readily interpretable and easy to understand but have three principal limitations. Firstly, they are unlikely to achieve as high a level of performance as a machine learning model derived from a set of features. Secondly, most of the handcrafted scores use parameters that are only available at least 24, if not 48 or 72 h after hospitalisation.^25^, ^50^ Thirdly, these scores were developed during the era of the original Atlanta classification that distinguished mild and severe AP, unlike the revised Atlanta classification that distinguishes mild, moderately severe and severe AP. As the predictive capabilities of these scores, comprehensively reviewed by Gurusamy et al.,^51^ have reached their limit, alternative approaches are needed.

In one of the earliest works using machine learning, Pearce et al.^35^ applied kernel logistic regression to predict the severity of AP using eight variables (age, arterial pH, serum CRP, Glasgow Coma Scale, pO_2_ on air, respiratory rate, serum creatinine and white blood cell count) obtained from 265 patients. Their model achieved a 0.82 AUC score, while the AUC of the APACHE II score was only 0.74. Hong et al.^52^ developed a score predicting severe AP, including systemic inflammatory response syndrome, serum albumin, blood urea nitrogen and pleural effusion (SABP score), with an AUC of 0.875, higher than that of BISAP, APACHE II, HAPS, Glasgow score, Japanese severity score and CRP. However, relatively small databases were available for the calculation of these scores.

Qiu et al.^53^ used three machine learning models (SVM, logistic regression, neural network) to predict the risk of multiple organ failure in severe AP. The models were built on a relatively small dataset of 263 patients, and the three models’ AUC scores were between 0.832 and 0.840, while the AUC of the APACHE score was 0.814. They found haematocrit, kinetic time (thromboelastogram), interleukin‐6 and creatinine to have the greatest predictive power. Ding et al.^54^ used artificial neural networks and logistic regression for the early prediction of in‐hospital mortality in AP. The authors used 12 variables that were collected within 24 h of admission from 337 patients. The performance of the model was relatively low compared to the other works, with an AUC of the neural network at 0.769 and logistic regression at 0.607. Akshintala and Khashab^55^ recently described the application of artificial intelligence to AP prediction in pancreaticobiliary endoscopy, presenting a simple AI‐based AP risk prediction calculator and decision‐making tool. All these previous results derived from relatively small cohorts^11^ suggest the potential of machine learning models to improve upon handcrafted scores, an approach that we have exploited in our work. Our 0.81 ± 0.033 AUC value achieved in far larger populations matches those of the former works, and our model is improving further with use, as it is applied even more widely.

Last but not least, our model and web application make early prediction possible. Although the study design allowed 12 h for the inclusion of patients, the model will work with only a few parameters shortly after the diagnosis of AP.

### 4.1 Strength and limitations

There are several strengths of our model. (1) We have used a large international cohort for both model development and external validation. (2) The model is continuously improving with experience. (3) We also explain the prediction with the help of SHAP values, which helps physicians understand the decision of the machine learning model. Moreover, it may also educate patients in finding how to change their lifestyle or behaviour to avoid developing AP again. (4) Our model uses only data that are available at the time of patient admission to the hospital, hence provides a very early prediction of the likelihood of severe AP. (5) Finally, we developed a web application, which for a given set of input parameters returns three outputs: the predicted severity score of AP, the confidence of the model, and the explanation of the prediction that highlights the key factors affecting the severity of AP.

The principal limitation of this study was imposed by its design, namely, the use of binary classification for non‐severe and severe AP to derive the model. Binary classification has enabled derivation of the likelihood of the development of severe AP but may not be able to accurately distinguish likelihoods of mild from moderately severe AP as these were entered as one class. This results in a score that is more akin to the original rather than revised Atlanta classification, although there may be limitations in the scores obtained for patients with local complications but without persistent organ failure. While the score calculated for any patient varies between 0 and 1, it may be easier for clinicians to understand percentage likelihoods instead; this feature can be altered in the future. More subtly, the confidence limits for the prediction of severity are wider moving away from the prediction spectrum endpoints, that is, with scores closer to the middle of the range. Nevertheless, our model is improving with experience; thus, these limitations might decrease with the use of the web application and feeding the model with further data. A further limitation of the study was that we could not exclude those patients with organ failure on admission.

### 4.2 Implications for practice

Based on the predictions, we can identify patients at increased risk for severe AP; therefore, the model can assist in early triage to intensive care units and the selection of patients for specific interventions. The easy‐to‐use web application (http://easy‐app.org/) is a useful tool for clinicians for early prediction. The more they use this application, the better the model becomes.

## 5 CONCLUSION

The EASY prediction score is a practical tool for identifying patients at a greater risk for severe AP within 24 h of hospital admission. The easy‐to‐use web application is available for clinicians and contributes to the improvement of the model.

## CONSENT TO PARTICIPATE

Written informed consent was obtained from all participants before enrolment.

## CONSENT FOR PUBLICATION

Not applicable.

## CONFLICT OF INTEREST

The authors do not have any conflicts of interest to declare.

## Supporting information

Supplementary material
