# Supplementary material for: EASY‐APP: An artificial intelligence model and application for early and easy prediction of severity in acute pancreatitis
Source: Clin Transl Med. 2022 Jun 2;12(6):e842. doi: 10.1002/ctm2.842 (PMC9162438; doi:10.1002/ctm2.842)
Supplement: Supplementary file 1 — Supplementary material [file CTM2-12-e842-s001.pdf]

Supplementary material

EASY-APP: An artificial intelligence model and application for early and easy prediction of severity in acute pancreatitis

HUNGARIAN PANCREATIC STUDY GROUP CONTRIBUTORS

Adrienn Halász[1,2], Veronika Dunás-Varga[1], Tamás Takács[3], László Czakó[3], Zoltán Szepes[3], Melania Macarie[4], Petr Pencik[5], Goran Poropat[6], Davor Stimac[6], Imanta Ozola-Zalite[7], Andrey Litvin[8], Árpád Patai[9], Kristina Zadorozhna[10], Árpád V. Patai[11], István Hritz[11], Elena Stilidi[12], János Novák[13], Masayasu Horibe[14], Georgiana Robu[15], László Lakatos[16], Ali Tüzün Ince[17], Gabriele Capurso[18], Dušan Leško[19], Dóra Illés[3], Dániel Pécsi[20], Péter Varjú[20], Klementina Ocskay[20,21], Márk Félix Juhász[20,22], Mária Földi[22,23], Alexandra Mikó[20,24], and Zsolt Szakács[20,25]

[1]Szent György Teaching Hospital of County Fejér, 1st Department of Internal Medicine, Székesfehérvár, Hungary
[2]Doctoral School of Clinical Medicine, University of Szeged, Szeged
[3]Department of Medicine, University of Szeged, Szeged, Hungary
[4]County Emergency Clinical Hospital of Târgu Mures - Gastroenterology Clinic and University of Medicine, Pharmacy, Sciences and Technology ”George Emil Palade”, Targu Mures, Romania
[5]Centrum péče o zažívací trakt, Vítkovická nemocnice a.s., Ostrava, Czech Republic
[6]Clinical Hospital Center Rijeka, Rijeka, Croatia
[7]Gastroenterology, Hepatology and Nutritional Centre, Pauls Stradins Clinical University Hospital, Riga, Latvia
[8]Immanuel Kant Baltic Federal University, Kaliningrad, Russia, Gomel Regional Clinical Hospital, Gomel, Belarus
[9]Markusovszky University Teaching Hospital, Szombathely, Hungary
[10]Bogomolets National Medical University, Kiev, Ukraine
[11]Department of Surgery, Transplantation and Gastroenterology, Semmelweis University, Budapest, Hungary
[12]Hospital of Medical Academy named after SI Georgievsky, Simferopol, Russia
[13]Pándy Kálmán Hospital of Békés County, Gyula, Hungary
[14]Division of Gastroenterology and Hepatology, Department of Internal Medicine, Keio University School of Medicine, Tokyo, Japan
[15]Central Military Emergency Hospital "Dr Carol Davila", Bucharest, Romania
[16]Centre of Internal Medicine, Csolnoky Ferenc Hospital, Veszprém, Hungary
[17]Hospital of Bezmialem Vakif University, School of Medicine, Istanbul, Turkey
[18]Digestive and Liver Disease Unit, S. Andrea Hospital University "Sapienza", Rome, Italy
[19]1st Department of Surgery, Pavol Jozef Šafárik University, Košice, Slovakia
[20]Institute for Translational Medicine, Medical School, University of Pécs, Pécs, Hungary
[21]Centre for Translational Medicine, Semmelweis University, Budapest, Hungary
[22]Heim Pál National Pediatric Institute
[23]Centre for Translational Medicine, Department of Medicine, University of Szeged, Szeged, Hungary
[24]Department of Medical Genetics, Medical School, University of Pécs, Pécs
[25]First Department of Medicine, Medical School, University of Pécs, Pécs, Hungary

Contributions: AH, VDV, TT, LC, ZS, MM, PP, GP, DS, IOZ, AL, ÁP, KZ, ÁVP, IH, ES, JN, MH, GR, LL, ATI, GC, and DL contributed to the data collection. DI, DP, PV, KO, MFJ, MF, AM, and ZS contributed to the assurance of data quality.

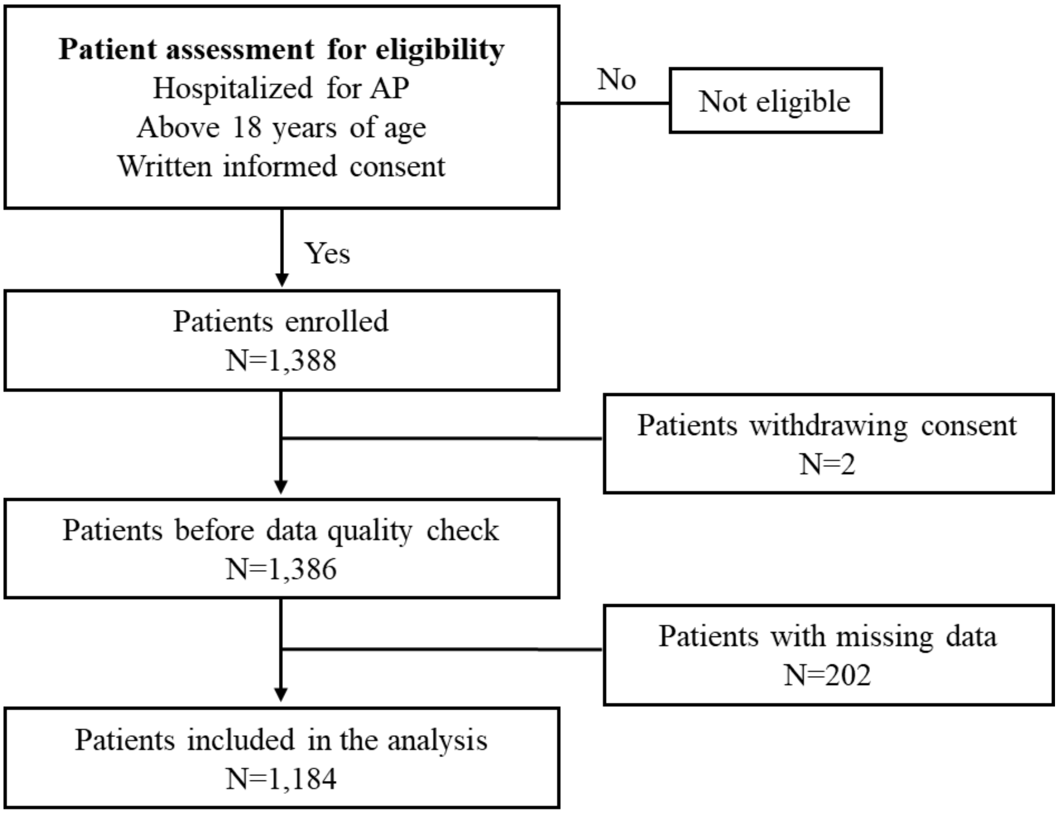

Supplementary figure 1. Flowchart of patient inclusion in the EASY study.

CENTRES OF THE ORIGINAL COHORT OF THE EASY STUDY

**EASY study - Institutes**			**Case no.**
First Department of Medicine, University of Pécs	Pécs	Hungary	584
Department of Internal Medicine, University of Debrecen	Debrecen	Hungary	149
Szent György University Teaching Hospital of County Fejér	Székesfehérvár	Hungary	93
Department of Medicine, University of Szeged	Szeged	Hungary	84
County Emergency Clinical Hospital of Târgu Mures	Targu Mures	Romania	76
Vilnius University Hospital Santariskiu Klinikos	Vilnius	Lithuania	31
General Surgery, Consorci Sanitari del Garraf	Sant Pere de Ribes	Spain	30
Helsinki University Central Hospital	Helsinki	Finland	30
Saint Luke Clinical Hospital	St. Petersburgh	Russia	30
Centrum Péče o Zažívací trakt, Vítkovická Nemocnice A.S.	Ostrava	Czech Republic	13
Clinical Hospital Centre Rijeka	Rijeka	Croatia	11
Gastroenterology, Hepatology and Nutritional Centre, Pauls Stradins Clinical University Hospital	Riga	Latvia	9
Gomel Regional Clinical Hospital	Gomel	Belarus	9
Markusovszky University Teaching Hospital	Szombathely	Hungary	9
Bogomolets National Medical University	Kiev	Ukraine	6
Second Department of Medicine, Semmelweis University	Budapest	Hungary	6
Medical Academy named after SI Georgievsky	Simferopol	Russia	5
Pándy Kálmán Hospital of County Békés	Gyula	Hungary	4
Keio University	Tokyo	Japan	2
Central Military Emergency Hospital "Dr Carol Davila"	Bucharest	Romania	1
Csolnoky Ferenc Hospital	Veszprém	Hungary	1
Jahn Ferenc South-Pest Hospital	Budapest	Hungary	1
**Total**			**1,184**

Supplementary table 1. Centres of the original cohort of the EASY study.

FURTHER EXAMPLES OF PREDICTIONS

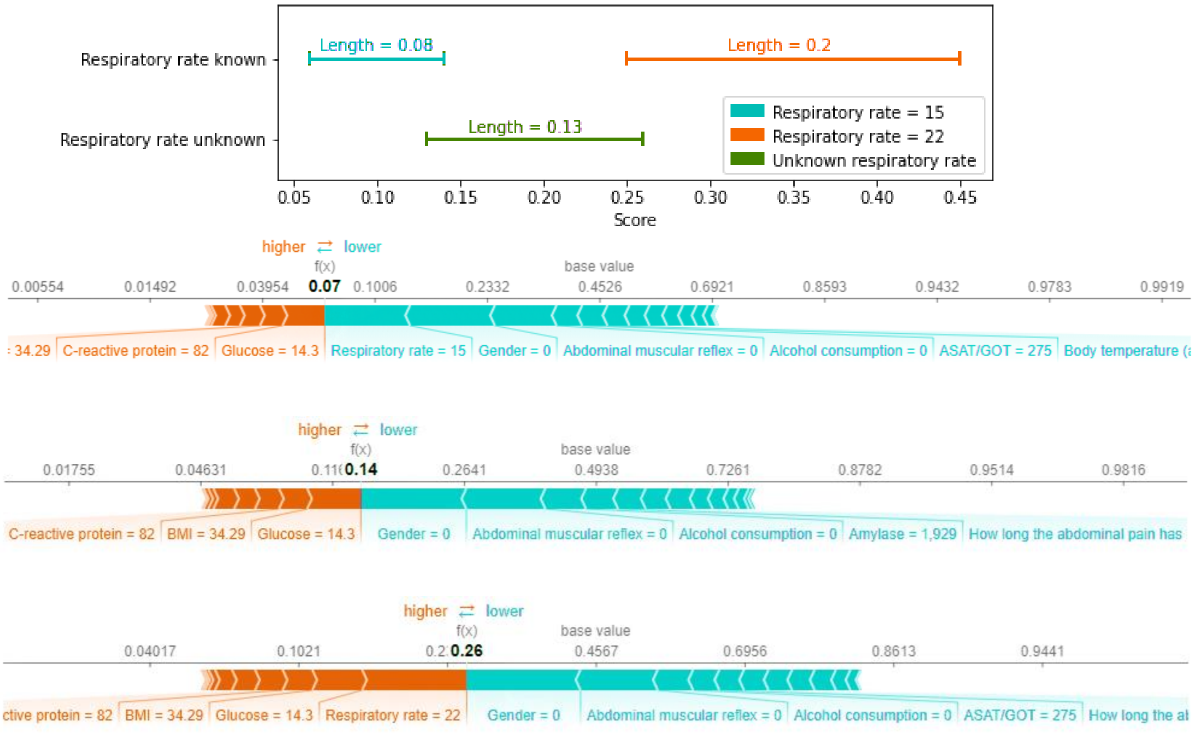

Supplementary figure 2. Further examples of predictions: the top plot shows the predicted severity score and its confidence interval with a low, high, and unknown value of respiratory rate, the lower figures show the corresponding local explanations.

Supplementary figure 1. shows the effect on the predicted severity score and its confidence when the value of the most important feature, the respiratory rate is low (respiratory rate = 15), high (respiratory rate = 22), and unknown (the other features are unchanged). The bottom part of the figure shows the three corresponding explanations of the predictions. From the figure, it is apparent, that the low respiratory rate pushes the predicted severity score lower (prediction = 0.07), and in this case, the confidence of the model is also higher (length of the confidence interval is 0.08). On the other hand, when the respiratory rate is relatively high, the predicted severity score also increases from 0.07 to 0.26, and the respiratory rate is 22 is the most influential negative factor that pushes the severity score lower. When the respiratory rate is unknown, both the predicted severity score and its confidence interval lies between the previous values.

Supplementary material

CHARACTERISTICS OF THE VALIDATION COHORT

INSTITUTES			No. of cases
Alicante University General Hospital, Alicante University	Alicante	Spain	1 655
Liverpool University Hospitals, University of Liverpool	Liverpool	UK	647
Hospital Val D'Hebron, University of Barcelona	Barcelona	Spain	454
The 4th Medical Clinic, "Iuliu Hatieganu" University of Medicine and Pharmacy	Cluj Napoca	Romania	408
**Total**			**3 164**

Supplementary table 2. Institutes of the validation cohort.

ALICANTE			Data Quality*
**Demographic data**			
Gender, male %	53.8%	female/male	100%
Age, mean (SD); min, max	64.5 (17.3)	[19, 100]	100%
BMI, mean (SD); min, max	27.4 (4.7)	[16.0, 53.9]	94%
**Anamnestic data**			
Alcoholic AP consumption, yes %	15.2%	yes/no	100%
Smoking, yes %	22.2%	yes/no	100%
Length of abdominal pain, mean (SD) in hours; min, max	no data		
**Admission data**			
Abdominal guarding, yes %	no data		
Abdominal tenderness, yes %	no data		
Body temperature (axillary), °C mean (SD); min, max	36.3 (0.7)	[34.0, 40.0]	98%
Systolic blood pressure (Hgmm), mean (SD); min, max	no data		
Diastolic blood pressure (Hgmm), mean (SD); min, max	no data		
Heart rate, mean (SD); min, max	82 (18)	[35, 160]	97%
Respiratory rate, mean (SD); min, max	18 (4)	[8, 40]	73%
**Laboratory parameters**			
Amylase (U/l), mean (SD); min, max	no data		
Aspartate transaminase (AST) (U/l), mean (SD); min, max	187.6 (242.0)	[4.0, 2122.0]	82%
Serum ionized Calcium (mmol/l), mean (SD); min, max	2.3 (0.2)	[1.3, 4.0]	60%
C-reactive protein (mg/l), mean (SD); min, max	no data		
Creatinine (µmol/l), mean (SD); min, max	102.3 (59.2)	[53.4, 884.0]	56%
Glucose (mmol/l), mean (SD); min, max	7.7 (3.6)	[1.0, 32.6]	99%
Potassium (mmol/l), mean (SD); min, max	4.1 (0.6)	[2.0, 7.0]	57%
Sodium (mmol/l), mean (SD); min, max	138.2 (3.8)	[115.0, 152.0]	99%
Urea (carbamide) (mmol/l), mean (SD); min, max	6.8 (3.8)	[0.5, 42.3]	99%
White blood cell count, mean (SD); min, max	11.5 (6.0)	[1.0, 38.7]	99%
**Outcome**			
The severity of acute pancreatitis, severe %	7.5%	non-severe/severe	100%

Supplementary table 3. Summary of the dataset from Alicante.

**LIVERPOOL**			**Data quality***
**Demographic data**			
Gender, male %	48.8%	female/male	100%
Age, mean (SD); min, max	55.4 (18.1)	[18, 96]	99%
BMI, mean (SD); min, max	no data		
**Anamnestic data**			
Alcoholic AP consumption, yes %	no data		
Smoking, yes %	no data		
Length of abdominal pain, mean (SD) in hours; min, max	22.1 (32.7)	[1, 168]	86%
**Admission data**			
Abdominal guarding, yes %	no data		
Abdominal tenderness, yes %	no data		
Body temperature (axillary), °C mean (SD); min, max	36.7 (0.5)	[34.0, 41.0]	99%
Systolic blood pressure (Hgmm), mean (SD); min, max	135.1 (26.2)	[59, 224]	100%
Diastolic blood pressure (Hgmm), mean (SD); min, max	79.1 (15.9)	[21, 186]	100%
Heart rate, mean (SD); min, max	83.3 (19-9)	[45, 165]	100%
Respiratory rate, mean (SD); min, max	17.7 (3.3)	[12, 46]	99%
**Laboratory parameters**			
Amylase (U/l), mean (SD); min, max	1471.9 (1057.0)	[33.0, 6303.0]	91%
Aspartate transaminase (AST) (U/l), mean (SD); min, max	no data		
Serum ionized Calcium (mmol/l), mean (SD); min, max	2.3 (0.1)	[1.4, 2.9]	62%
C-reactive protein (mg/l), mean (SD); min, max	33.4 (71.0)	[1.0, 529.0]	71%
Creatinine (μmol/l), mean (SD); min, max	86.0 (55.7)	[8.1, 694.0]	98%
Glucose (mmol/l), mean (SD); min, max	7.6 (4.2)	[2.1, 71.0]	67%
Potassium (mmol/l), mean (SD); min, max	4.2 (0.5)	[2.2, 7.6]	92%
Sodium (mmol/l), mean (SD); min, max	138.2 (3.8)	[120.0, 159.0]	98%
Urea nitrogen (carbamide) (mmol/l), mean (SD); min, max	5.8 (4.7)	[1.1, 65.0]	98%
White blood cell count, mean (SD); min, max	13.4 (5.5)	[1.4, 68.0]	97%
**Outcome**			
The severity of acute pancreatitis, severe %	8.8%	non-severe/severe	100%

Supplementary table 4. Summary of the dataset from Liverpool.

**BARCELONA**			**Data quality***
**Demographic data**			
Gender, male %	51.3%	female/male	100%
Age, mean (SD); min, max	64.9 (18.5)	[17, 98]	100%
BMI, mean (SD); min, max	28.2 (5.3)	[16.4, 55.3]	98%
**Anamnestic data**			
Alcoholic AP consumption, yes %	16.9%	yes/no	94%
Smoking, yes %	24.9%	yes/no	97%
Length of abdominal pain, mean (SD) in hours; min, max	41.4 (61.8)	[1, 360]	100%
**Admission data**			
Abdominal guarding, yes %	37.6%	yes/no	97%
Abdominal tenderness, yes %	7.5%	yes/no	97%
Body temperature (axillary), °C mean (SD); min, max	36.4 (0.7)	[34.0, 38.6]	99%
Systolic blood pressure (Hgmm), mean (SD); min, max	132.9 (24.6)	[60, 207]	100%
Diastolic blood pressure (Hgmm), mean (SD); min, max	72.3 (15.1)	[30, 167]	100%
Heart rate, mean (SD); min, max	82.3 (18.0)	[40, 148]	100%
Respiratory rate, mean (SD); min, max	16.5 (3.2)	[12, 40]	95%
**Laboratory parameters**			
Amylase (U/l), mean (SD); min, max	1219.4 (1424.9)	[6.0, 20420.0]	99%
Aspartate transaminase (AST) (U/l), mean (SD); min, max	236.0 (321.9)	[8.0, 3515.0]	100%
Serum ionized Calcium (mmol/l), mean (SD); min, max	2.3 (0.2)	[1.3, 2.9]	98%
C-reactive protein (mg/l), mean (SD); min, max	55.7 (80.3)	[0.3, 437.7]	99%
Creatinine (µmol/l), mean (SD); min, max	88.4 (43.3)	[30.9, 321.8]	100%
Glucose (mmol/l), mean (SD); min, max	8.3 (3.6)	[3.2, 35.4]	100%
Potassium (mmol/l), mean (SD); min, max	3.9 (0.5)	[2.5, 5.7]	99%
Sodium (mmol/l), mean (SD); min, max	137.0 (3.7)	[116.3, 154.8]	100%
Urea nitrogen (carbamide) (mmol/l), mean (SD); min, max	16.1 (9.1)	[2.2, 72.1]	100%
White blood cell count, mean (SD); min, max	13.5 (5.6)	[2.3, 45.5]	100%
**Outcome**			
The severity of acute pancreatitis, severe %	11.7%	non-severe/severe	100%

Supplementary table 5. Summary of the dataset from Barcelona.

CLUJ NAPOCA			Data quality*
**Demographic data**			
Gender, male %	51.0%	female/male	100%
Age, mean (SD); min, max	60.1 (16.5)	[21, 93]	100%
BMI, mean (SD); min, max	no data		
**Anamnestic data**			
Alcoholic AP consumption, yes %	18.9%	yes/no	100%
Smoking, yes %	no data		
Length of abdominal pain, mean (SD) in hours; min, max	58.0 (63.2)	[2, 504]	77%
**Admission data**			
Abdominal guarding, yes %	11.5%	yes/no	100%
Abdominal tenderness, yes %	92.6%	yes/no	100%
Body temperature (axillary), °C mean (SD); min, max	no data		
Systolic blood pressure (Hgmm), mean (SD); min, max	138.0 (23.7)	[60, 231]	98%
Diastolic blood pressure (Hgmm), mean (SD); min, max	77.5 (13.7)	[30, 150]	98%
Heart rate, mean (SD); min, max	81.9 (16.9)	[50, 150]	97%
Respiratory rate, mean (SD); min, max	no data		
**Laboratory parameters**			
Amylase (U/l), mean (SD); min, max	804.6 (844.3)	[10.5, 5349.0]	100%
Aspartate transaminase (AST) (U/l), mean (SD); min, max	173.9 (259.8)	[8.0, 3481.0]	100%
Serum ionized Calcium (mmol/l), mean (SD); min, max	2.3 (0.2)	[1.0, 3.5]	88%
C-reactive protein (mg/l), mean (SD); min, max	82.4 (93.8)	[0.6, 523.0]	98%
Creatinine (μmol/l), mean (SD); min, max	96.0 (69.9)	[30.1, 534.1]	100%
Glucose (mmol/l), mean (SD); min, max	7.6 (3.9)	[2.6, 39.8]	99%
Potassium (mmol/l), mean (SD); min, max	3.9 (0.6)	[2.6, 7.2]	100%
Sodium (mmol/l), mean (SD); min, max	135.2 (3.5)	[124.0, 145.0]	100%
Urea nitrogen (carbamide) (mmol/l), mean (SD); min, max	16.3 (12.1)	[1.1, 93.9]	100%
White blood cell count, mean (SD); min, max	12.8 (5.7)	[1.3, 39.4]	100%
**Outcome**			
The severity of acute pancreatitis, severe %	17.2%	non-severe/severe	100%

Supplementary table 6. Summary of the dataset from Cluj Napoca.

VALIDATION ANALYSIS

1. Training set: Original EASY cohort, test set: data from Liverpool, Cluj Napoca, Alicante, Barcelona.

In the first part of the validation phase, we used our whole EASY database as a training set, and other different studies were used as test sets, i.e., the machine learning model was trained on the EASY cohort, and then we tested its performance on the other international datasets separately (Liverpool, Barcelona, Cluj Napoca, and Alicante). After cleaning and processing the data, we analysed them. A significant problem was that these studies were created for other purposes instead of the severity prediction of pancreatitis. Thus, many parameters were missing, which made harder the analysis, and decreased the AUC values.

Data from Liverpool study group: In the case of this study the record of BMI, abdominal tenderness, abdominal guarding, GOT values were missing. These parameters were not recorded because of the design of this study protocol. As Supplementary figure 2. shows, the ROC curve has a lower AUC score on the Liverpool data (0.77±0.040), compared to the EASY cohort (0.81±0.033).

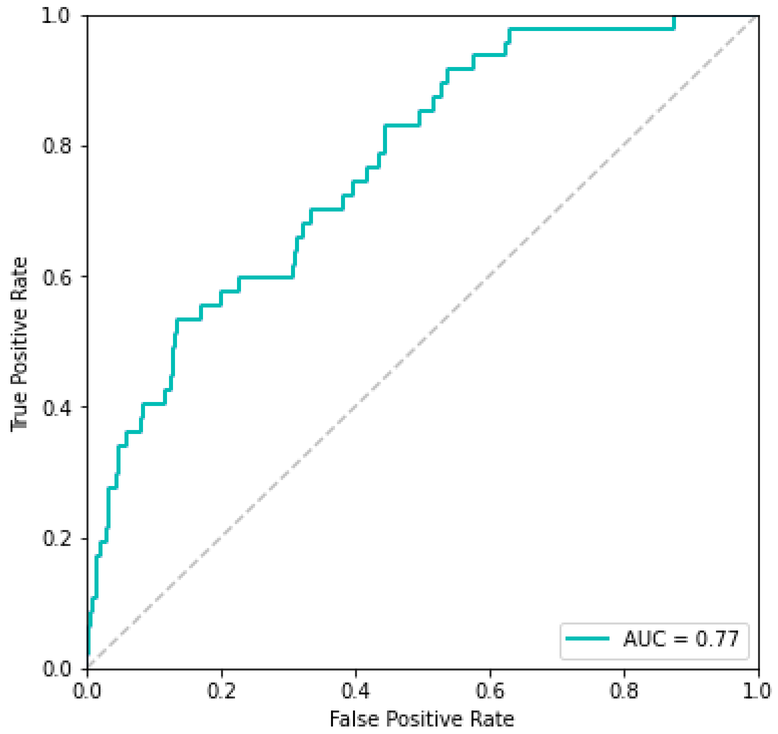

Supplementary figure 3. The ROC curve of the model that was trained on the EASY data and evaluated on the Liverpool data.

In the case of the data from Barcelona, the ROC curve has a similar AUC score (0.79 ±0.039) to the EASY cohort, as it can be seen in Supplementary figure 3.

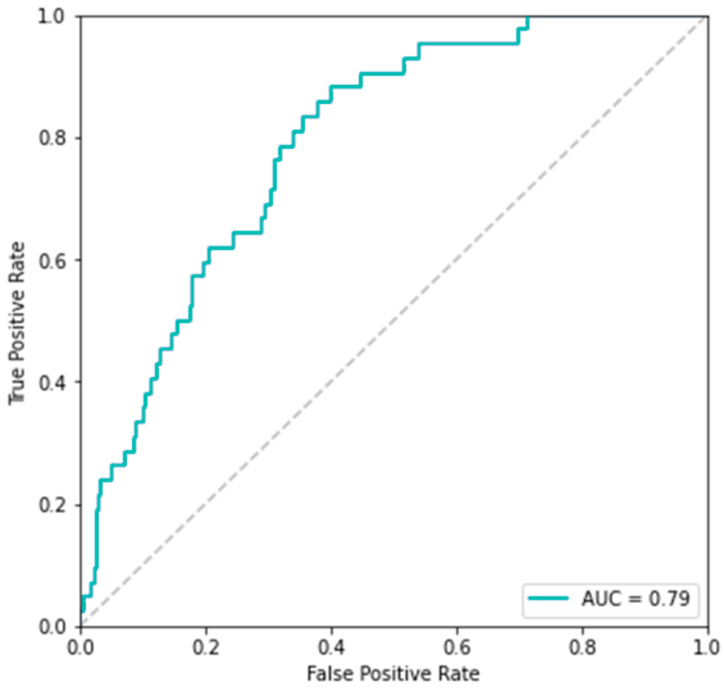

Supplementary figure 4. The ROC curve of the model that was trained on the EASY data and evaluated on the Barcelona data.

In the case of the Cluj Napoca data, for 90% of the patients, BMI, respiratory rate, body temperature, and smoking habit parameters were missing, which caused a significant problem, because BMI and body temperature are very important predictive factors of severe AP.

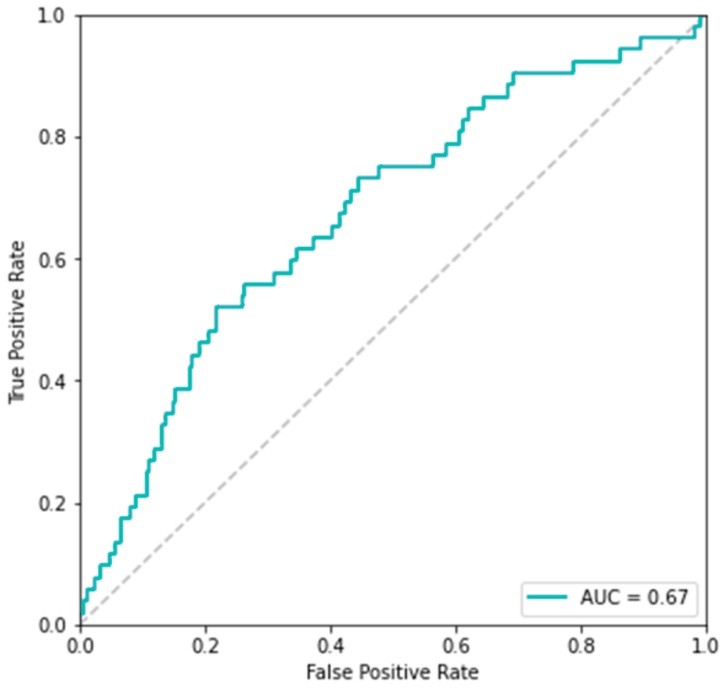

Supplementary figure 5. The ROC curve of the model that was trained on the EASY data and evaluated on the Cluj Napoca data.

That is one of the reasons why the ROC curve has a poor 0.67 ±0.044 AUC score (Supplementary figure 4). Another possible reason for the low AUC score, is that we have found that the joint distribution of serum carbamide and amylase is different on the EASY and the Cluj-Napoca data in the case of severe AP patients (Supplementary figure 5.). Maybe this phenomenon was caused by the different populations.

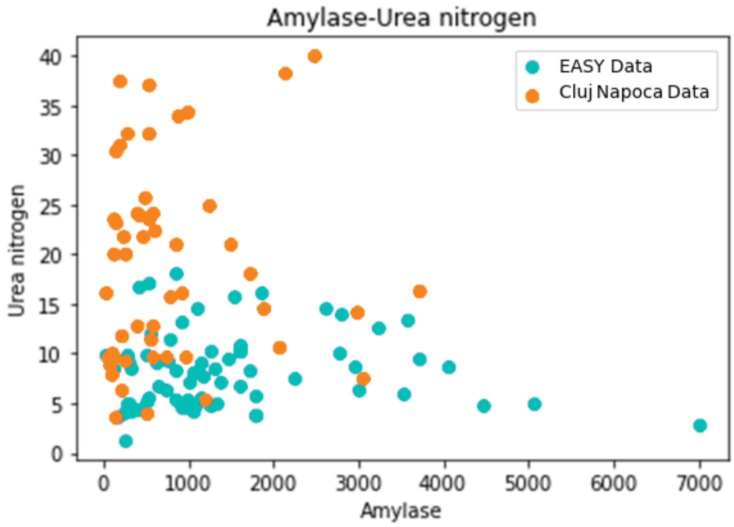

Supplementary figure 6. Joint distribution of serum carbamide and amylase in the severe cases in the EASY and Cluj Napoca dataset.

As Supplementary figure 6. illustrates, without serum amylase and carbamide parameters, the ROC curve improved significantly with an AUC score of 0.74 ±0.041.

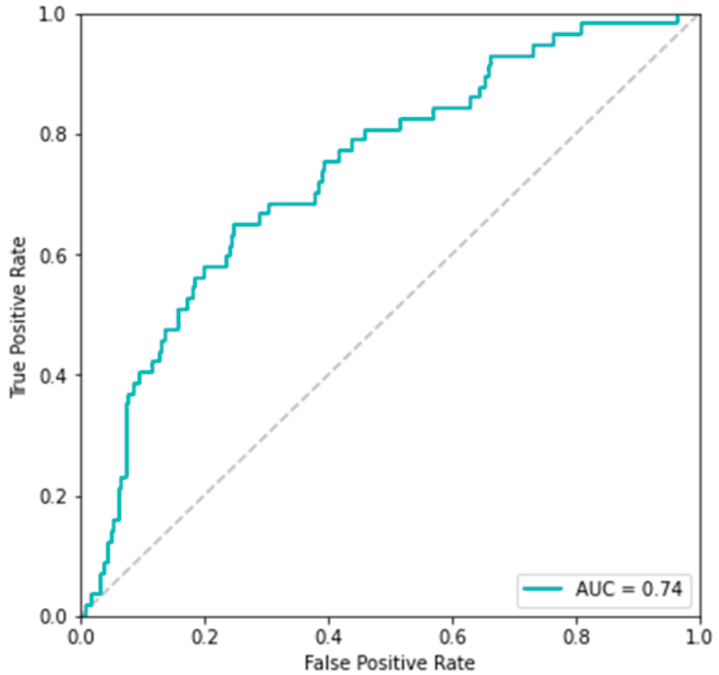

Supplementary figure 7. The ROC curve of the model that was trained on the EASY data and evaluated on the Cluj Napoca data without serum amylase and carbamide parameters.

Finally, we evaluated our model (which was trained on the EASY cohort) on the Atlantis data (Alicante study group), this dataset also showed a lower AUC score of 0.72 ±0.036 (Supplementary figure 7), because of the lack of CRP, abdominal pain duration time, systolic/diastolic blood pressure, abdominal tenderness, abdominal guarding, serum amylase values.

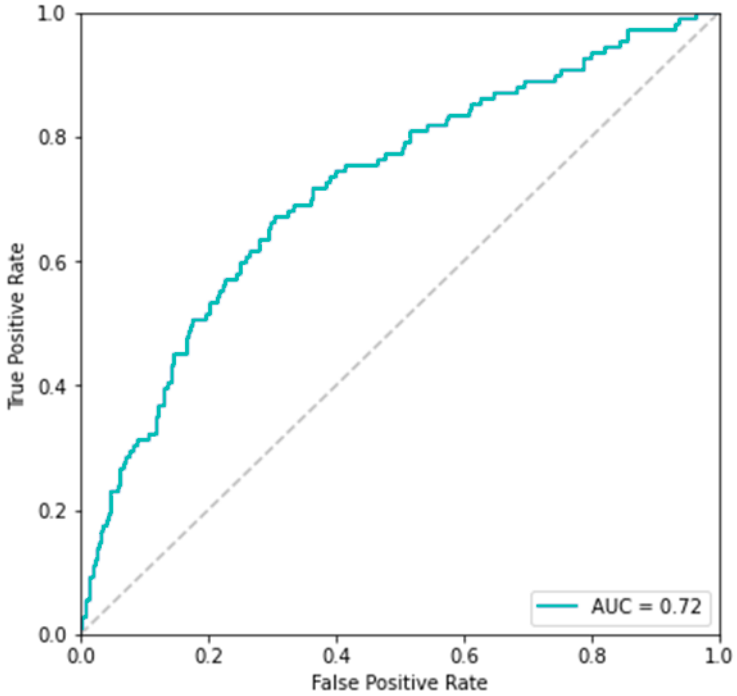

Supplementary figure 8. The ROC curve of the model that was trained on the EASY data and evaluated on the Atlantis data.

2. Training and also test set: data from Liverpool, Cluj Napoca, Alicante, Barcelona.

Centres	Liverpool (n=647)	Barcelona (n=454)	Cluj Napoca (n=408)	Alicante (n=1655)
AUC	0.749 ±0.048	0.782 ±0.037	0.760 ±0.038	0.779 ±0.032

Supplementary table 7. Cross-validated AUC of models trained on the corresponding dataset.

Supplementary table 7. shows the performance (cross-validated AUC) of the model when we train and evaluate it separately on the other international datasets.

In what follows, we study how the performance of the model increases if the training set contains observations from different datasets. The new centres' data were divided into equally sized subsets , and then x% of these subsets were used as a training data set together with the whole EASY data. The rest of the data (the other subsets) were used for validation, i.e., to measure the performance of the model. This process is repeated in a cross-validation manner. The results are detailed in Supplementary table 8.. From the table it is apparent, that the performance of the model increases with more training

data. In other words, the more data we add (from other centres) to the EASY cohort for training, the higher the AUC score of the model on the remaining test data set is.

Training set EASY+ x % of the given study	Liverpool AUC (n=647)	Barcelona AUC (n=454)	Cluj Napoca AUC (n=408)	Alicante AUC (n=1655)
0%	0.772 ±0.040	0.790 ±0.039	0.736 ±0.041	0.718 ±0.036
33%	0.773 ±0.037	0.792 ±0.037	0.780 ±0.036	0.764 ±0.032
50%	0.776 ±0.037	0.791 ±0.035	0.785 ±0.034	0.777 ±0.031
67%	0.780 ±0.036	0.803 ±0.034	0.784 ±0.034	0.786 ±0.031
80%	0.781 ±0.034	0.804 ±0.033	0.793 ±0.032	0.791 ±0.029

Supplementary table 8. Cross-validated AUC scores of the model trained on the union of the EASY data complemented with x% of the corresponding international dataset..

ROC curves with cross-validation.

Finally, we evaluate the model's performance using cross-validation as follows. We select one of the cohorts on which we test the model. Then we divide the data into 4 equally sized subsets. The data of the other cohorts and three subsets of the selected cohort are used for training and the remaining one subset of the selected cohort is used to measure the model's performance (AUC).

Supplementary figure 8., 9., 10., 11., and 12. show the cross-validated ROC curve and the corresponding AUC score of the model on the EASY, Liverpool, Barcelona, Cluj Napoca, and Alicante respectively. The difference between the 'A' and 'B' figures, is that in the case of the 'A' figures, the model was trained and tested separately on the different cohorts, and in the 'B' figures, the training set was supplemented with the other cohorts in the aforementioned way.

The result of the 4-fold cross-validation on the EASY cohort, where the training datasets (3 folds, i.e., 75% of the EASY data) were supplemented with the Liverpool, Cluj Napoca, Alicante, and Barcelona data.

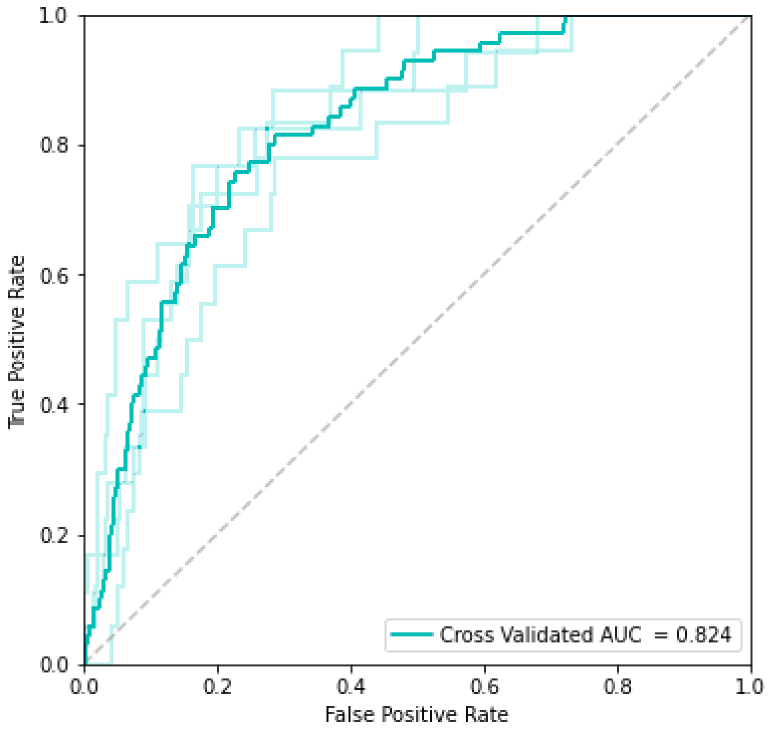

Supplementary figure 9. The result of the 4-fold cross-validation on the EASY cohort, where the training datasets (75% of the EASY data) were supplemented with the Liverpool, Cluj Napoca, Alicante, and Barcelona data.

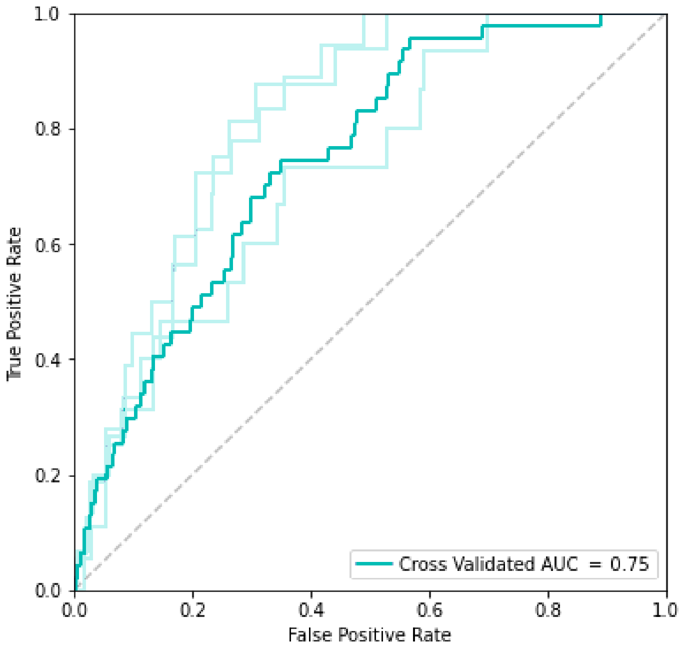

Supplementary figure 10.A. The result of the 4-fold cross-validation on the Liverpool dataset.

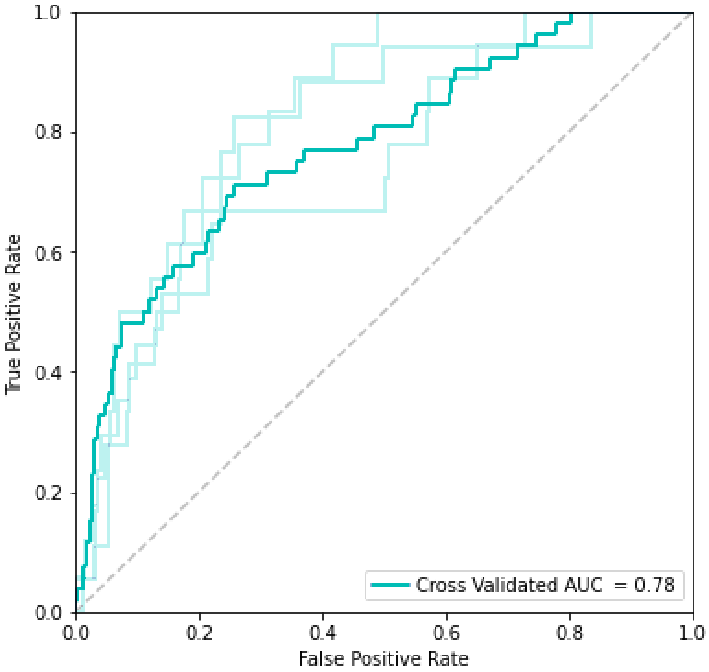

Supplementary figure 10.B. The result of the 4-fold cross-validation on the Liverpool cohort, where the training datasets (75% of the Liverpool data) were supplemented with the EASY, Cluj Napoca, Alicante, and Barcelona data.

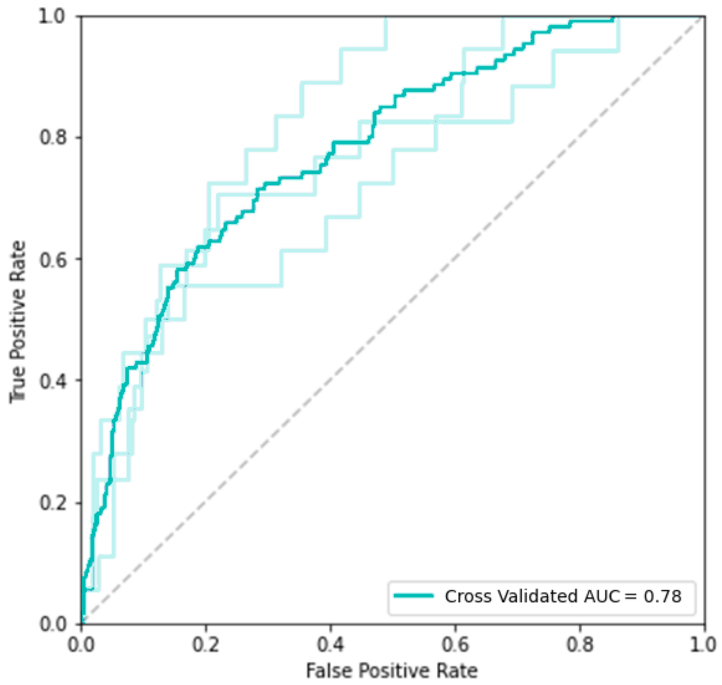

Supplementary figure 11.A. The result of the 4-fold cross-validation on the Barcelona dataset.

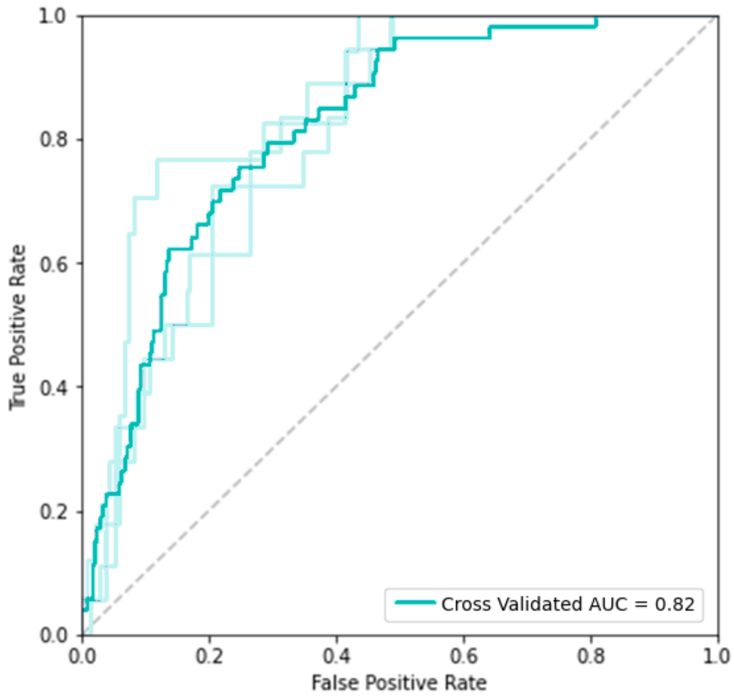

Supplementary figure 11.B. The result of the 4-fold cross-validation on the Barcelona cohort, where the training datasets (75% of the Barcelona data) were supplemented with the Cluj Napoca, Liverpool, Alicante, and EASY data.

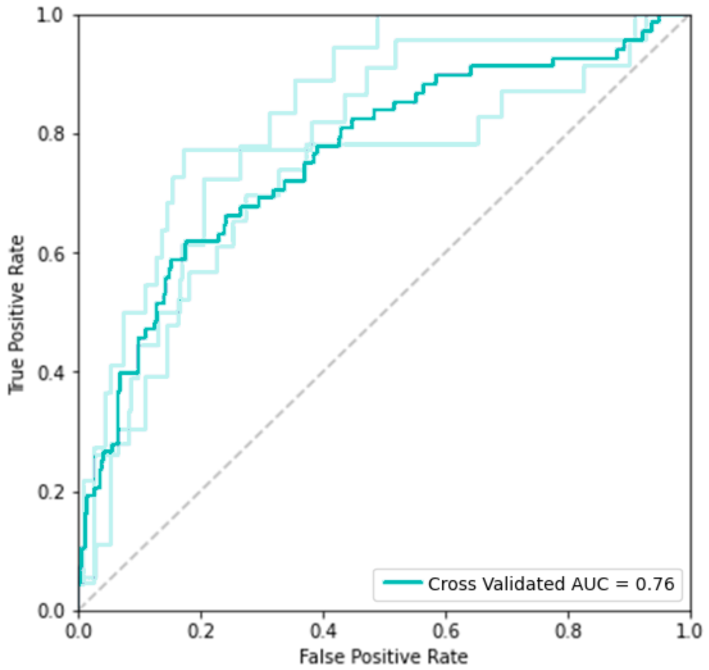

Supplementary figure 12.A. The result of the 4-fold cross-validation on the Cluj Napoca dataset.

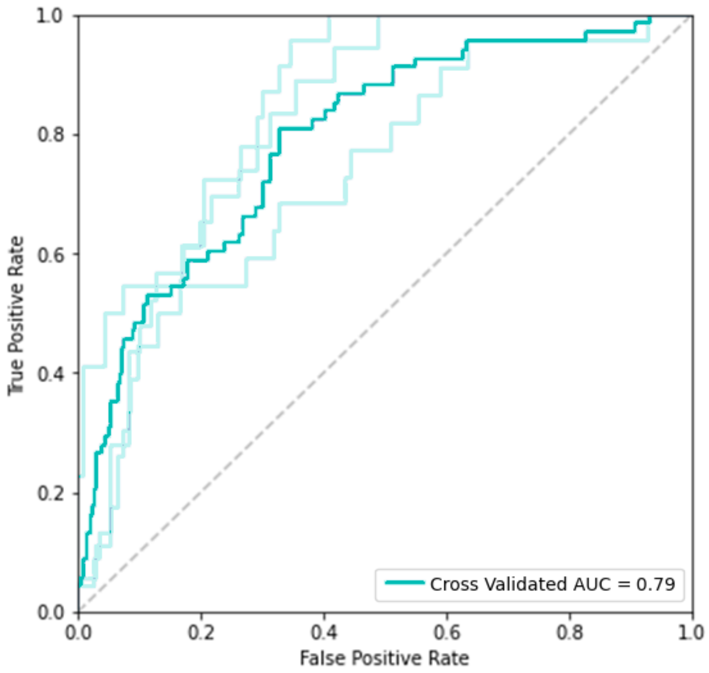

Supplementary figure 12.B. The result of the 4-fold cross-validation on the Cluj Napoca cohort, where the training datasets (75% of the Cluj Napoca data) were supplemented with the Barcelona, Liverpool, Alicante, and EASY data.

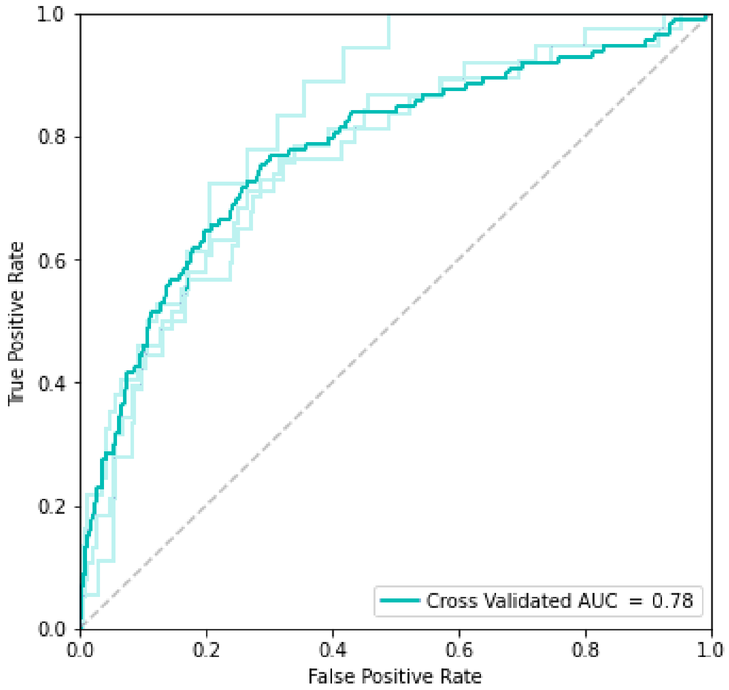

Supplementary figure 13.A. The result of the 4-fold cross-validation on the Alicante dataset.

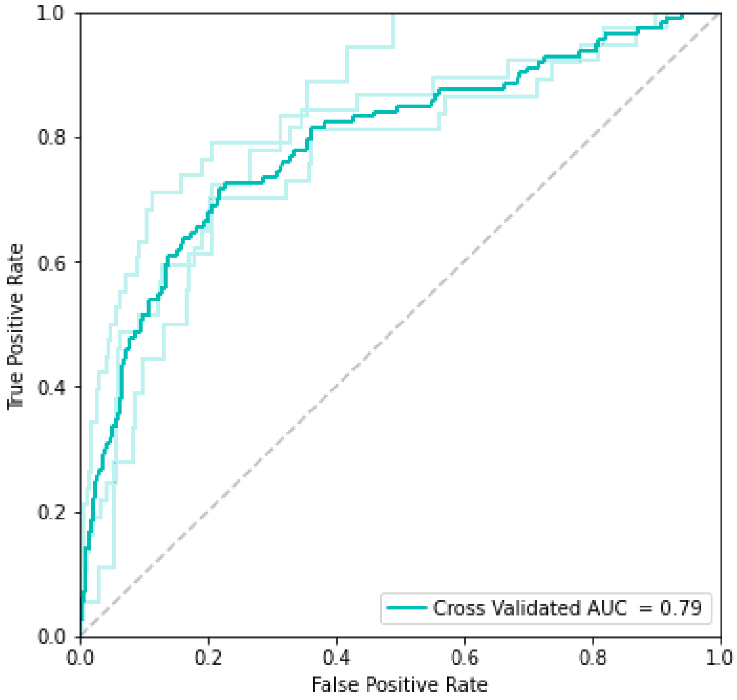

Supplementary figure 13.B. The result of the 4-fold cross-validation on the Alicante cohort, where the training datasets (75% of the Alicante data) were supplemented with the Barcelona, Liverpool, Cluj Napoca, and EASY data.

3. The model is cross-validated on the union of the five different cohorts (Alicante, Barcelona, Cluj Napoca, EASY, Liverpool).

The results of the 4-fold cross-validation on the whole dataset (union of all data sets) can be seen on Supplementary figure 13. The figure suggests that the performance of the model improves when we train the model on the union of all the datasets. In this case the cross-validated AUC score is 0.803, however, when we train the model separately then the average cross-validated AUC score is only 0.776.

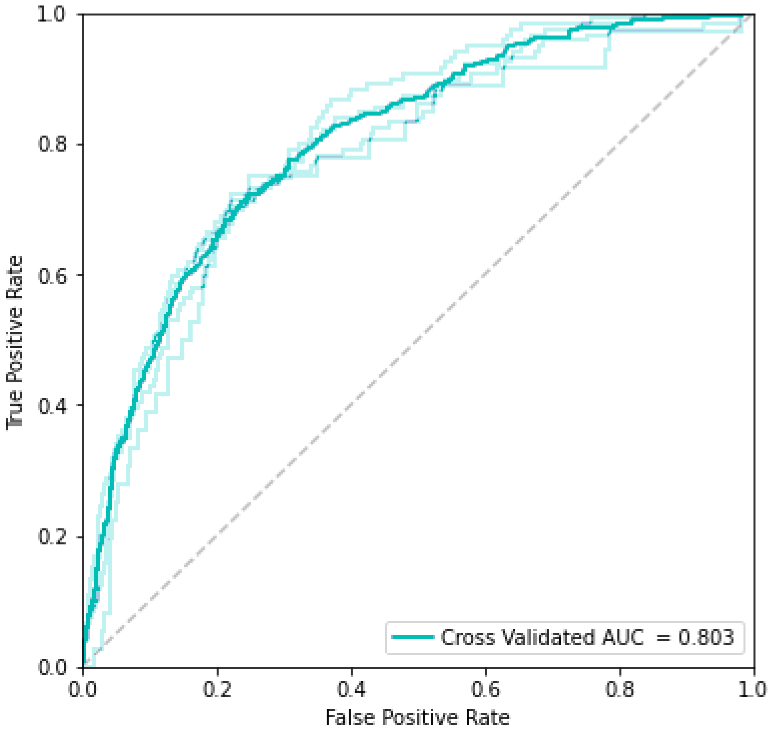

Supplementary figure 14. The result of the 4-fold cross-validation on the whole dataset together.

4. The most influential features on different cohorts

The SHAP importance of the variables were also validated on the five different cohorts. We studied how the most influential predictors vary if the model is trained on different cohorts. In Supplementary table 9 we can see the rank of the most influential variables with respect to different cohorts. While there is some between-cohort variation in the ranking of the features, we can observe that the most important predictors are highly ranked in all cohorts.

SHAP feature importance rank	EASY	Liverpool	Barcelona	Cluj-Napoca	Alicante	Mean rank
Creatinine	**5.**	**1.**	**1.**	**1.**	**8.**	3.2
Glucose	**4.**	**3.**	**3.**	**7.**	**3.**	4
Respiratory rate	**1.**	**5.**	11.	missing	**1.**	4.5
Urea Nitrogen	**6.**	**2.**	**2.**	missing (after preprocessing)	**9.**	4.75
White blood cell count	**10.**	**4.**	**5.**	**5.**	**2.**	5.2
Gender	**3.**	**6.**	**7.**	**6.**	**7.**	5.8
Abdominal muscular reflex	**2.**	missing	**8.**	**8.**	missing	6
Calcium	17.	14.	12.	**2.**	**5.**	10
Age	12.	**7.**	**4.**	15.	13.	10.2
C-reactive protein	11.	**9.**	18.	**4.**	missing	10.5
Pulse	19.	16.	**6.**	**3.**	**10.**	10.8
Body Temperature	14.	13.	16.	missing	**4.**	11.75

Supplementary table 9. Top 12 features based on their average SHAP feature importance rank using all cohorts

EASY-APP AVAILABILITY AND REGISTRATION

The web application is available at: http://easy-app.org/

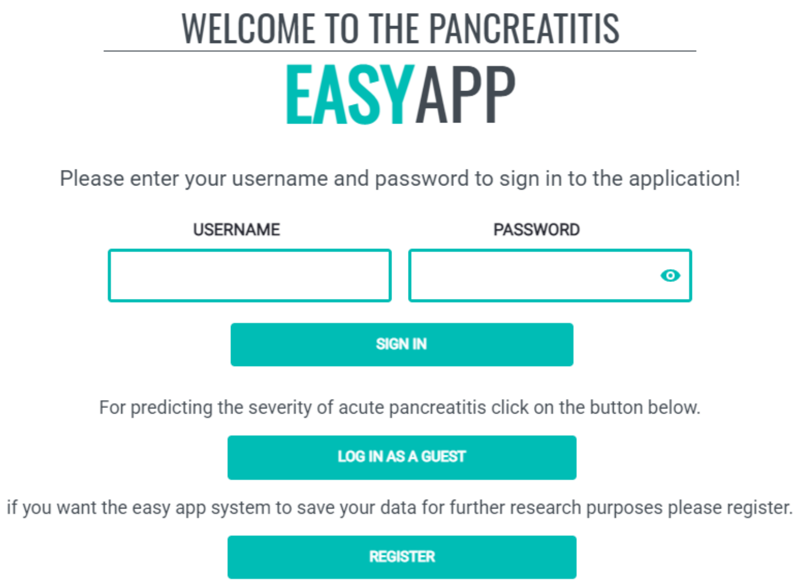

The model calculates a numerical probability value between 0 and 1. The higher the number, the higher the risk for severe acute pancreatitis. Together with the numerical values, a textual interpretation is given as well. Upon request, the application provides the confidence interval in addition to the numerical value. For educational purposes, with the help of the SHAP values, the explanation of the prediction highlighting the key factors affecting the severity of AP is shown too. Built-in validations filter out invalid values.

The application can be used in two ways. The prediction is possible without registration, however, in the case of registration, the data and the prediction will be stored and the given prediction contributes to the development of the model.

TRIPOD Checklist: Prediction Model Development and Validation

Section/Topic			Checklist Item	Page
**Title and abstract**				
Title	1	D;V	Identify the study as developing and/or validating a multivariable prediction model, the target population, and the outcome to be predicted.	1
Abstract	2	D;V	Provide a summary of objectives, study design, setting, participants, sample size, predictors, outcome, statistical analysis, results, and conclusions.	2
**Introduction**				
Background and objectives	3a	D;V	Explain the medical context (including whether diagnostic or prognostic) and rationale for developing or validating the multivariable prediction model, including references to existing models.	3
	3b	D;V	Specify the objectives, including whether the study describes the development or validation of the model or both.	4
**Methods**				
Source of data	4a	D;V	Describe the study design or source of data (e.g., randomized trial, cohort, or registry data), separately for the development and validation data sets, if applicable.	4
	4b	D;V	Specify the key study dates, including start of accrual; end of accrual; and, if applicable, end of follow-up.	4
Participants	5a	D;V	Specify key elements of the study setting (e.g., primary care, secondary care, general population) including number and location of centres.	4
	5b	D;V	Describe eligibility criteria for participants.	4
	5c	D;V	Give details of treatments received, if relevant.	4
Outcome	6a	D;V	Clearly define the outcome that is predicted by the prediction model, including how and when assessed.	5
	6b	D;V	Report any actions to blind assessment of the outcome to be predicted.	not applicable
Predictors	7a	D;V	Clearly define all predictors used in developing or validating the multivariable prediction model, including how and when they were measured.	5
	7b	D;V	Report any actions to blind assessment of predictors for the outcome and other predictors.	not applicable
Sample size	8	D;V	Explain how the study size was arrived at.	8
Missing data	9	D;V	Describe how missing data were handled (e.g., complete-case analysis, single imputation, multiple imputation) with details of any imputation method.	6
Statistical analysis methods	10a	D	Describe how predictors were handled in the analyses.	6
	10b	D	Specify type of model, all model-building procedures (including any predictor selection), and method for internal validation.	6-7
	10c	V	For validation, describe how the predictions were calculated.	9
	10d	D;V	Specify all measures used to assess model performance and, if relevant, to compare multiple models.	9-11
	10e	V	Describe any model updating (e.g., recalibration) arising from the validation, if done.	14+ supplementary
Risk groups	11	D;V	Provide details on how risk groups were created, if done.	not applicable
Development vs. validation	12	V	For validation, identify any differences from the development data in setting, eligibility criteria, outcome, and predictors.	14+ supplementary
**Results**				
Participants	13a	D;V	Describe the flow of participants through the study, including the number of participants with and without the outcome and, if applicable, a summary of the follow-up time. A diagram may be helpful.	8-9
	13b	D;V	Describe the characteristics of the participants (basic demographics, clinical features, available predictors), including the number of participants with missing data for predictors and outcome.	8-9
	13c	V	For validation, show a comparison with the development data of the distribution of important variables (demographics, predictors and outcome).	8-9
Model development	14a	D	Specify the number of participants and outcome events in each analysis.	8-9
	14b	D	If done, report the unadjusted association between each candidate predictor and outcome.	not applicable
Model specification	15a	D	Present the full prediction model to allow predictions for individuals (i.e., all regression coefficients, and model intercept or baseline survival at a given time point).	not applicable
	15b	D	Explain how to the use the prediction model.	13-14
Model performance	16	D;V	Report performance measures (with CIs) for the prediction model.	9-10
Model-updating	17	V	If done, report the results from any model updating (i.e., model specification, model performance).	14+ supplementary
**Discussion**				
Limitations	18	D;V	Discuss any limitations of the study (such as nonrepresentative sample, few events per predictor, missing data).	15-16
Interpretation	19a	V	For validation, discuss the results with reference to performance in the development data, and any other validation data.	14+ supplementary
	19b	D;V	Give an overall interpretation of the results, considering objectives, limitations, results from similar studies, and other relevant evidence.	14-17
Implications	20	D;V	Discuss the potential clinical use of the model and implications for future research.	13-16
**Other information**				
Supplementary information	21	D;V	Provide information about the availability of supplementary resources, such as study protocol, Web calculator, and data sets.	13-16
Funding	22	D;V	Give the source of funding and the role of the funders for the present study.	17

*Items relevant only to the development of a prediction model are denoted by D, items relating solely to a validation of a prediction model are denoted by V, and items relating to both are denoted D;V. We recommend using the TRIPOD Checklist in conjunction with the TRIPOD Explanation and Elaboration document.
